# Novel induction of CD40 expression by tumor cells with RAS/RAF/PI3K pathway inhibition augments response to checkpoint blockade

**DOI:** 10.1186/s12943-021-01366-y

**Published:** 2021-06-06

**Authors:** Chi Yan, Nabil Saleh, Jinming Yang, Caroline A. Nebhan, Anna E. Vilgelm, E. Premkumar Reddy, Joseph T. Roland, Douglas B. Johnson, Sheau-Chiann Chen, Rebecca L. Shattuck-Brandt, Gregory D. Ayers, Ann Richmond

**Affiliations:** 1 Department of Veterans Affairs, Tennessee Valley Healthcare System, 432 PRB, 2220 Pierce Ave, Nashville, TN 37232 USA; 2 Department of Pharmacology, Vanderbilt University School of Medicine, Nashville, TN USA; 3 Department of Medicine, Division of Hematology and Oncology, Vanderbilt University Medical Center, Nashville, TN USA; 4 Department of Pathology, The Ohio State University, Columbus, OH USA; 5 Department of Oncological Sciences, Icahn School of Medicine at Mount Sinai, New York, NY USA; 6 Departments of Surgery and Pediatrics and the Epithelial Biology Center, Vanderbilt University School of Medicine, Nashville, TN USA; 7 Department of Biostatistics, Vanderbilt University Medical Center, Nashville, TN USA

**Keywords:** RAS/RAF/PI3K, CD40, Immunogenic cell death, Immune checkpoint blockade, Melanoma

## Abstract

**Background:**

While immune checkpoint blockade (ICB) is the current first-line treatment for metastatic melanoma, it is effective for ~ 52% of patients and has dangerous side effects. The objective here was to identify the feasibility and mechanism of RAS/RAF/PI3K pathway inhibition in melanoma to sensitize tumors to ICB therapy.

**Methods:**

Rigosertib (RGS) is a non-ATP-competitive small molecule RAS mimetic. RGS monotherapy or in combination therapy with ICB were investigated using immunocompetent mouse models of BRAF^wt^ and BRAF^mut^ melanoma and analyzed in reference to patient data.

**Results:**

RGS treatment (300 mg/kg) was well tolerated in mice and resulted in ~ 50% inhibition of tumor growth as monotherapy and ~ 70% inhibition in combination with αPD1 + αCTLA4. RGS-induced tumor growth inhibition depends on CD40 upregulation in melanoma cells followed by immunogenic cell death, leading to enriched dendritic cells and activated T cells in the tumor microenvironment. The RGS-initiated tumor suppression was partially reversed by either knockdown of CD40 expression in melanoma cells or depletion of CD8^+^ cytotoxic T cells. Treatment with either dabrafenib and trametinib or with RGS, increased CD40^+^SOX10^+^ melanoma cells in the tumors of melanoma patients and patient-derived xenografts. High CD40 expression level correlates with beneficial T-cell responses and better survival in a TCGA dataset from melanoma patients. Expression of CD40 by melanoma cells is associated with therapeutic response to RAF/MEK inhibition and ICB.

**Conclusions:**

Our data support the therapeutic use of RGS + αPD1 + αCTLA4 in RAS/RAF/PI3K pathway-activated melanomas and point to the need for clinical trials of RGS + ICB for melanoma patients who do not respond to ICB alone.

**Trial registration:**

NCT01205815 (Sept 17, 2010).

**Graphical abstract:**

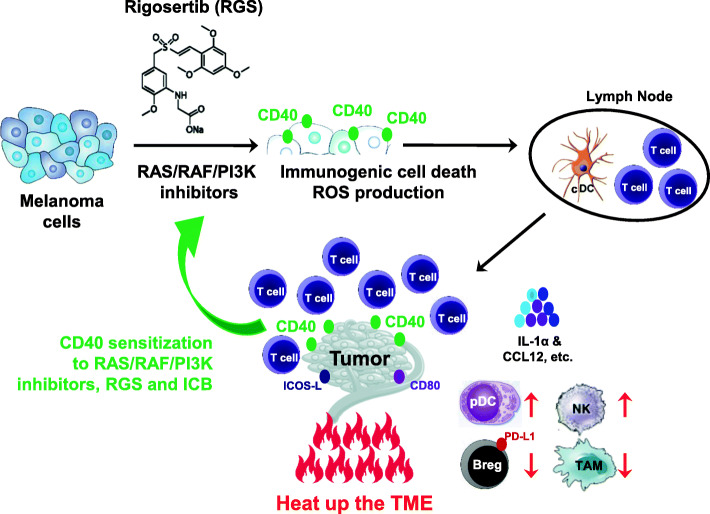

**Supplementary Information:**

The online version contains supplementary material available at 10.1186/s12943-021-01366-y.

## One-sentence summary

RAS/RAF/PI3K inhibition promotes CD40 expression by melanoma cells, induces immunogenic cell death, and enhances response to immune checkpoint inhibitors.

## Introduction

Approximately 20% of melanoma patient tumors harbor mutations in the neuroblastoma RAS viral oncogene homolog (NRAS), 60% have a mutually exclusive mutation to NRAS in the *v-raf* murine sarcoma viral oncogene homolog B1 (BRAF), and 31% have mutation in the phosphoinositide 3-kinase (PI3K) pathway [1, 2]. These pathways are crucial to support melanoma cell proliferation, survival, or evasion of cell death. While BRAF^V600mut^ patients with unresectable or metastatic melanoma respond well to inhibitors of BRAF combined with MEK inhibitors [3, 4], a majority of patients will develop acquired resistance to these drugs through myriad of resistance mechanisms [5–7]. Recent advances in immune checkpoint blockade (ICB) therapy, such as targeting programmed cell death protein 1 (PD-1)/PD-ligand 1 (PD-L1) and/or cytotoxic T-lymphocyte-associated protein 4 (CTLA-4), illustrate the power of enhancing the patient’s endogenous anti-melanoma immune responses [4, 8–10]. Anti-PD-1 combined with α-CTLA4 immunotherapy significantly prolong survival for > 52% of melanoma patients with previously untreated advanced melanoma (> 60 months median overall survival [OS]). Moreover, 44% of melanoma patients treated with α-PD-1 alone (19.9 months median OS) exhibit extended survival (36.9 months median OS) [10]. Currently, ICB is a first-line therapy for unresectable metastatic melanoma. However, many patients do not respond and adverse events can be severe and often deadly [11]. Therefore, improved targeted therapeutic approaches are needed, especially after patients have progressed on immunotherapy or targeted therapy alone.

For melanoma patients whose tumors harbor mutation in *NRAS*, treatment with BRAF inhibitors is contraindicated. However, MEK inhibitors alone or in combination with CDK4/6 inhibitors, or PI3K-AKT pathway inhibitors, resulted in partial responses in phase I/II clinical trials [12–14]. Rigosertib (RGS), also known as ON01910.Na, a non-ATP-competitive small molecule RAS mimetic, has the potential to block RAS-RAF-MEK-ERK and PI3K-AKT-mTOR signaling pathways and to interfere with CRAF interaction with polo-like kinase 1 (PLK1) [15–17]. While multiple mechanisms of action of RGS have been reported, all studies have found it to be a potent inhibitor of tumor growth. RGS inhibits tumor growth in xenografts of colorectal and lung cancer and blocks tumor growth of a transgenic model of pancreatic cancers induced by K-RAS^G12D^ expression [18]. In head and neck squamous cell carcinoma, RGS inhibits the PI3K/mTOR pathway, blocks cell cycle and induces cytotoxicity through oxidative stress-induced reactive oxygen species (ROS) and activation of ERK/JNK pathways, resulting in increased phosphorylation and cytoplasmic translocation of ATF-2 [19]. In diffuse large B-cell lymphoma, RGS is reported to sequester the sumoylated c-MYB/TRAF2 complex in the cytoplasm, resulting in G1-cell cycle arrest and apoptosis [20]. Several phase I and phase II/III clinical trials have now been performed with RGS. These trials report modest toxicity, including trials in patients with myelodysplastic syndrome (MDS) [21], high-risk MDS after DNA methyltransferase inhibitor therapy [22], ovarian cancer [23], metastatic pancreatic cancer (with or without gemcitabine) [24], and advanced cancers [25]. Melanoma has not yet been a focus for clinical trials with RGS. Given the potential for NRAS/BRAF/PI3K mutant melanoma to be particularly sensitive to RGS, it is important to evaluate whether RGS is effective in preclinical models for the treatment of melanoma. Here we have linked response to RGS to expression of CD40 by melanoma tumor cells.

CD40, a cell surface molecule of the tumor necrosis factor (TNF) receptor family, was first identified on antigen-presenting cells (e.g., B cells, macrophages and dendritic cells [DC]), and later shown to be expressed on the other cell types, including hematopoietic progenitors, platelets, eosinophils, T cells, epithelial cells, endothelial cells, fibroblasts and tumor cells [26]. In melanoma, CD40 is generally expressed by 30–50% human melanocytic lesions and melanoma cell lines [27]. Proinflammatory cytokines, such as interferon-gamma (IFNγ) and tumor necrosis factor-alpha (TNFα), and epigenetic modulators, such as histone deacetylase (HDAC) inhibitors, can induce CD40 expression on melanoma cells [27, 28]. Importantly, the induction of CD40 on cancer cells, but not normal cells, promotes apoptotic and/or necrotic signaling, which results in the cell death of renal cell carcinoma [29], urothelial cell carcinoma [30], ovarian carcinoma [31, 32], cervical carcinoma [33] and bladder carcinoma [34]. Besides the direct cytotoxic effects in cancer cells, the ligation of CD40 on human melanoma cells also modulates tumor immunogenicity through upregulation of the expression of major histocompatibility complex (MHC) molecules and the production of proinflammatory factors (e.g., IL-6, IL-8 and TNFα, etc.) [35]. Furthermore, CD40 was shown to co-stimulate both anti-CD3-triggered CD4^+^ and CD8^+^ human T cells [35] and enhance melanoma cell susceptibility to T cell lysis [28]. However, the regulation and function of CD40 in melanoma cells and its prognostic value in treatment responses remain to be examined.

Here, we report that RGS suppresses PI3K-mediated AKT^T308^ and mTORC2-mediated AKT^Ser473^ phosphorylation, increases CD40 expression and induces caspase3-dependent melanoma cell death. RGS is well tolerated and effective in immunocompetent mouse melanoma models YUMM3.3 (BRAF^mut^) and B16F10 (BRAF^WT^). RGS significantly increases the frequency of plasmacytoid DC (pDC) and conventional DC (cDC) subsets in the tumor microenvironment (TME) and tumor-draining lymph node (TDLN), respectively. In a dose-dependent manner, RGS favors the proliferation and activation of tumor-infiltrating T cells (especially CD8^+^ cytotoxic T cells [Tc]), and the induction of NK cells, along with a reduction in M2-like macrophages in the TME. The anti-tumor effect of RGS is substantially abrogated in athymic mice bearing either the murine melanoma tumors or patient-derived xenografts (PDX). Remarkably, RGS exhibits synergistic effects with the combination of αPD-1/αCTLA-4 ICB in B16F10 melanoma. At the mechanistic level, RGS monotherapy and in combination with ICB sensitizes tumors to immune activation via induced immunogenic cell death (ICD), associated with upregulation of costimulatory signals (such as CD40, CD80, and ICOS-L) on melanoma cells. We demonstrate here that RGS and BRAF/MEK inhibitors induce CD40 expression on murine melanoma cell lines and responsive patient melanoma cells in PDX models. Notably, we observe a significant induction of CD40^+^SOX10^+^ melanoma cells in the tumors of melanoma patients post BRAF inhibitor treatment by multiplex IHC analysis. An extensive search of human cancer databases indicates that the gain of CD40/CD80/ICOSL copy number in melanoma patients is associated with significantly better survival. We also find that the levels of CD40 in human melanoma cells correlate to the response to RAF inhibitor and ICB treatments. Together, our work reveals a novel role of RAS/RAF/PI3K inhibition in ICD via promoting CD40 expression on melanoma cells as well as in inducing an anti-tumor immune response. Importantly this work provides preclinical evidence for the combination of RGS and ICB treatment as systemic therapy in patients with unresectable or metastatic melanoma.

## Results

### Rigosertib induces melanoma cell death and inhibits melanoma tumor growth in vitro and in vivo

**Fig. 1:**
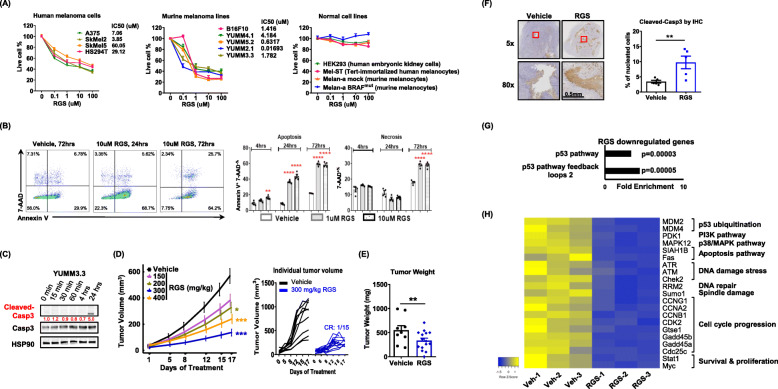
Rigosertib induces melanoma cell death and inhibits melanoma tumor growth in vivo. **a** IC50 determined by CellTilter Blue assay at 72 h post RGS treatment. The melanoma and normal cells were seeded in 96-well plate with 3000 cells per well. **b** Dot plots and quantified results of the Annexin V^+^ and 7-AAD^+^ cell percentage in YUMM3.3 culture with or without RGS treatment. **c** Whole-cell extracts were harvested and immunoblotted. HSP90 was used as a loading control for densitometry quantification (red numbers). **d**, **e** Tumor volume and weight of YUMM3.3 melanomas in C57BL/6 female mice. Daily RGS treatment starts at day 10 post tumor cell inoculation. CR, complete regression. **f** Representative images and quantitative results of cleaved-caspase3 protein levels observed from day 17 post RGS treatment (300 mg/kg) of YUMM3.3 tumors by immunohistochemistry. **g** Gene ontology enrichment analysis of gene targets downregulated in YUMM3.3 tumors post 17 days of RGS treatment (300 mg/kg). Results for Fold Enrichment > 3 and FDR *p* < 0.05 are displayed. **h** Heatmap summarizes 21 p53-associated targets from a total of 1495 genes screened that were significantly downregulated due to RGS treatment. **a**-**f** pooled data obtained from at least two different experiments (*n* = 4 ~ 15) are shown. **g**, **h** were triplicates

We first evaluated the cytotoxic activity of RGS in four human and six murine melanoma cell lines with diverse genetic backgrounds, including cells with *NRAS*^*Q61R*^, *BRAF*^*mut*^, *PTEN*^*null*^, *TP53*^*null/mut*^ and/or *CDKN2A*^*null*^ (Suppl.Table 1). Based on the CellTiter-Blue assay, the viability of all melanoma cell lines tested was significantly inhibited by micromolar concentrations of RGS (Fig. 1 a). Notably, while melanoma cells bearing either NRAS^Q61R^ (SKMel2) or BRAF^mut^ (HS294T, SkMel5, A375, YUMM 5.2, YUMM 2.1 and YUMM 3.3) were sensitive to low dose RGS treatment at 0.1 μM, the NRAS^WT^BRAF^WT^ B16F10 cells were not sensitive to 0.1 μM RGS, but were sensitive to 1 μM. By contrast, normal cells, including HEK293 cells and melanocytes from both human (Mel-ST) and murine (Melan-a with BRAF^WT^ or BRAF^mut^) origins, were totally refractory to RGS treatment. The dose-dependent cytotoxic effects of RGS on melanoma cells were further confirmed using crystal violet assay (Suppl.Fig. 1A). While BRAF^V600E^ is active independent of RAS binding, other signaling pathways that co-mediate melanoma growth remain dependent on RAS binding to the RAS-binding domain for activation. Given that BRAF^mut^ is the most common mutation in melanoma [36], we first focused on BRAF^mut^ melanoma extensively for the following experiments. Using flow cytometric analyses, we observed that RGS triggered a dose- and time-dependent induction of apoptosis and necrosis in BRAF^mut^ YUMM3.3 melanoma cells (Fig. 1 b).

Several studies have indicated that the ICD induction requires rapid generation of ROS and further ROS-based endoplasmic reticulum (ER) stress that contributes to antigen release and the induction of antitumor immunity [37]. We found that RGS promoted ROS production in both human SkMel5 and murine YUMM3.3 melanoma cells, and this effect reached a peak at 4 h post-treatment (Suppl.Fig. 1B). Interestingly, a prolonged ROS production (~ 24 h post-treatment) was observed in the highly sensitive YUMM3.3 cells (IC50 = 1.782 μM) as compared to the less sensitive SkMel5 cells (IC50 = 60.05 μM). It was previously reported that RGS-induced ROS accumulation mediates ERK/JNK cascade activation in HeLa cells and head and neck cancer cell lines [19, 38]. However, ERK/JNK activation levels were not induced in the melanoma cells in response to RGS (Suppl.Fig. 1C). Instead, RGS treatment rapidly (<15mins) suppressed phosphorylation of AKT^T308^ in SkMel5 and YUMM3.3 melanoma cells and AKT^Ser473^ in A375 and SkMel5 melanoma cells (Suppl.Fig. 1D). Consistent with the apoptosis assay results, there was a clear induction of cleaved-caspase3 in YUMM3.3 cells at 24 h post-RGS treatment (Fig. 1 c). In contrast, mitotic markers PLK1 and FOXM1 were rapidly and consistently reduced in SkMel5 melanoma cells after RGS treatment (Suppl.Fig. 1E). Together, these results demonstrate that RGS suppresses AKT activation, inhibits cell viability and promotes ICD (e.g., ROS production and apoptosis/necrosis) in melanoma cells.

To identify the optimal effective in vivo dose of RGS in the melanoma tumors, we conducted a dose-escalation experiment (0, 150, 200, 300, 400 mg/kg) on YUMM3.3 tumors in C57BL/6 mice (Fig. 1 d). Oral delivery of RGS resulted in significant dose-dependent inhibition of tumor volume after 17 days of treatment that reached a plateau at 300 and 400 mg/kg doses with comparable 60% inhibition of tumor volume. We also observed that one of 15 mice exhibited complete tumor regression in response to the treatment (Fig. 1 d). Significant inhibition of tumor weight occurred with RGS treatment at 300 mg/kg dose (Fig. 1 e).

**Fig. 2:**
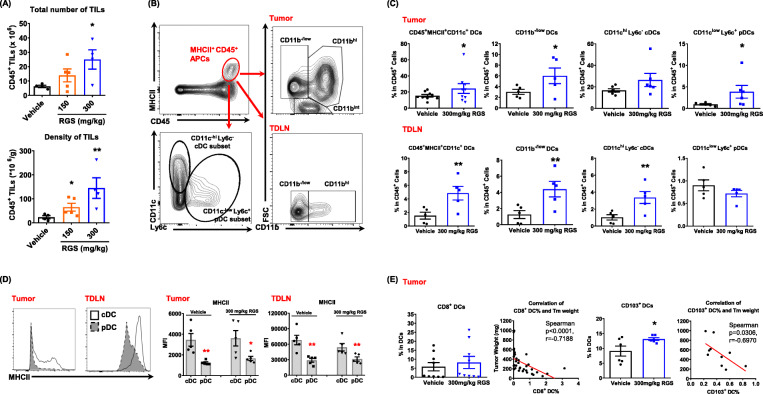
Rigosertib increases the frequency of dendritic cells in tumors and tumor-draining lymph nodes (TDLN). YUMM3.3 tumors and TDLN were collected 17 days post-treatment. **a** Total number and density of CD45^+^ leukocytes in tumors were obtained by flow cytometry. **b** Gating strategy for dendritic cells in tumors and TDLN. **c-e** Frequency and mean fluorescent intensity (MFI) of dendritic cell subsets and their correlation with tumor burden. Pooled data obtained from at least two different experiments (*n* = 5 ~ 10) are shown

To test whether RGS-induced anti-tumor effects are BRAF^mut^-specific or gender-dependent, we administrated RGS to female mice bearing NRAS^WT^BRAF^WT^ B16F10 tumors (Suppl.Fig. 2A), as well as to male mice bearing YUMM3.3 tumors (Suppl.Fig. 2B). Up to 60% of tumor growth inhibition was observed in both models. Similar to the in vitro observations (Fig. 1 c), the levels of cleaved-caspase3 were significantly increased in RGS-treated YUMM3.3 tumor sections compared to the vehicle control-treated counterparts (Fig. 1 f). Notably, the YUMM3.3 murine melanoma with constitutively active BRAF (RAS binding independent) was sensitive to RGS treatment. This indicates that in BRAF^mut^ melanoma the RGS-induced inhibitory effects may not be dependent on RAS binding to activate RAF, highlighting a role for RGS in the inhibition of other RAS-downstream signals (such as PI3K/AKT) that are dependent on RAS binding to RAS-binding domain (RBD) for activation.

**Fig. 3:**
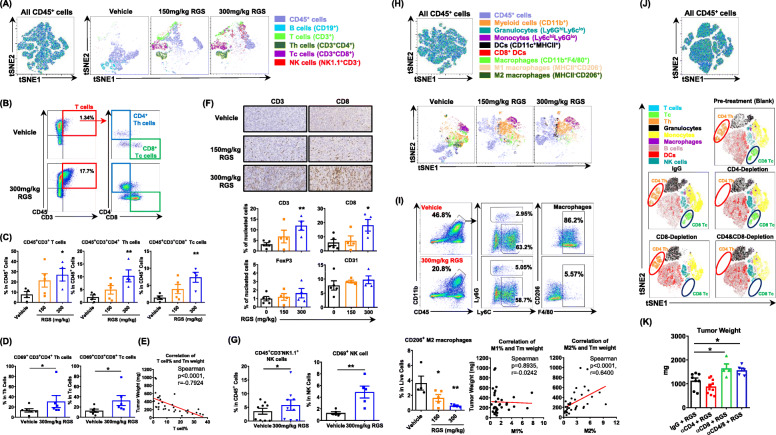
Rigosertib promotes T cell and NK cell responses but attenuates tumor-associated M2 macrophages in the tumor microenvironment. YUMM3.3 tumors were collected 17 days post-treatment. **a**, **h** Live CD45^+^ leukocytes were concatenated after downsampling to 20,000 events for subsequent high-dimensional data analysis to normalize the contribution among samples under different treatments. Samples were then analyzed in parallel by t-SNE and manually gated leukocyte populations were overlaid onto the total t-SNE map using FlowJo 10.5.3. **b-e**, **g**, **i** Flow cytometric and **f** IHC analysis of YUMM3.3 tumors at day 17 post-treatment. **j** Flow cytometric tSNE analysis of PBMC samples from YUMM3.3 tumor-bearing mice treated with RGS before and after CD4 and/or CD8 antibody depletion treatments. (K) Tumor weight (day 16 post RGS treatment) of YUMM3.3 tumors in C57BL/6 mice with and without CD4 and/or CD8 depletion. **a**-**i** pooled data obtained from at least two different experiments (*n* = 5 ~ 10) are shown. **j**, **k** were replicates (*n* = 10 per group)

To provide a more comprehensive assessment of RGS responses, we performed bulk RNA sequencing analysis on the YUMM3.3 tumors (RGS and vehicle treated), including tumor cells, stromal cells, and immune cells. RGS specifically down-regulated genes are involved in the p53 pathway (Fig. 1 g, h). Specifically, RGS treatment reduced the expression of genes encoding proteins involved in p53-ubiquitination, including *MDM2* and *MDM4*. In addition, GO Consortium pathway analysis indicated that these down-regulated genes function in the activation of PI3K-MAPK pathways, DNA damage/repair, spindle damage, cell cycle progression, as well as survival and proliferation. The data revealed increased expression of genes involved in the plasminogen activating cascade and synaptic vesicle trafficking in RGS-treated tumors (Suppl.Fig. 3). Query through the Vesiclepedia database (microvesicles.org) showed that the all of RGS-induced synaptic vesicle genes are encoding proteins that are located in either tumor microvesicles or exosomes. Interestingly, increased tumor vesicle formation has been linked to ICD [39].

### Rigosertib converts the immune environment of melanoma tumors from “cold” to “hot”

Having demonstrated a role for RGS in promoting melanoma cell death and suppressing melanoma tumor growth in vivo, we next questioned how RGS might shape the immune system in the TME and TDLNs. Immune profiling by flow cytometric analysis showed that RGS treatment of mice bearing YUMM3.3 tumors promoted an increase in the total number and density of tumor-infiltrating leukocytes (Fig. 2 a). To identify the immune populations responsible for the anti-tumor effects, we first subtyped the intratumoral DCs that are likely to respond to the tumor antigens released as a result of the RGS-induced ICD (Fig. 2 b). We observed that there was a significant increase of the tumor-infiltrating DCs (Fig. 2 c), specifically CD11b^−/low^CD11c^low^Ly6C^+^ plasmocytoid DCs (pDCs). Consistently, RGS also triggered the induction of CD11b^−/low^ DCs in the TDLN (Fig. 2 c). In contrast to the TME profile where CD11c^low^Ly6C^+^ pDCs were increased, RGS promoted the frequency of CD11c^hi^Ly6C^−^ conventional DCs (cDCs) in the TDLNs. We observed that cDC possess > 2-fold the level of MHCII on the cell surface as compared to pDCs of melanoma-bearing mice (Fig. 2 d). This suggests that increased cDC numbers in TDLN of RGS-treated mice may facilitate improved tumor antigen presentation; thus, the DC subset-specific induction matches well with the tissue-specific immune functions. Given that both lymphoid tissue-resident CD8^+^ and CD103^+^ immigrating DCs have excellent capacities to cross-present exogenous tumor antigens to activate CD8^+^ T cells [40], RGS treatment induced the frequency of these DC subsets in the TME and their levels are negatively correlated with tumor burden (Fig. 2 e).

**Fig. 4:**
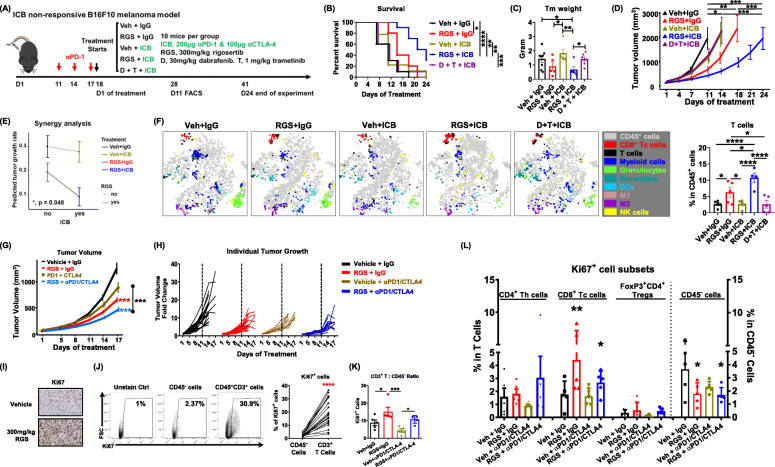
Rigosertib improves T-cell responses to αPD-1/αCTLA-4 therapy. **a** Schematic of ICB non-responsive B16F10 melanoma model. **b**-**d** Survival of C57BL/6 female mice, day 11 tumor weight post treatment and tumor volume of B16F10 tumors. Mice reached end point and sacrificed when tumors in the experiment exceeded 15 mm in diameter or became perforated. The experiment was terminated when no mice survived in groups other than the RGS + ICB group. **e** Analysis of synergy. Interaction plot for tumor growth rate with 95% confidence interval by treatment. **f** Live CD45^+^ leukocytes were concatenated after downsampling to ~ 12,500 events for t-SNE analysis through flow cytometry and T-cell frequency was shown. **g** Tumor volume of YUMM3.3 tumors in C57BL/6 female mice. Treatment starts at day 10 post tumor cell inoculation. **h** Spider plots of tumor volume changes overtime for individual YUMM3.3 tumors. **i** IHC and **j-l** Flow cytometric analysis of YUMM3.3 tumors at day 17 post-treatment. Data were replicates from one experiment (*n* = 10 per group)

**Fig. 5:**
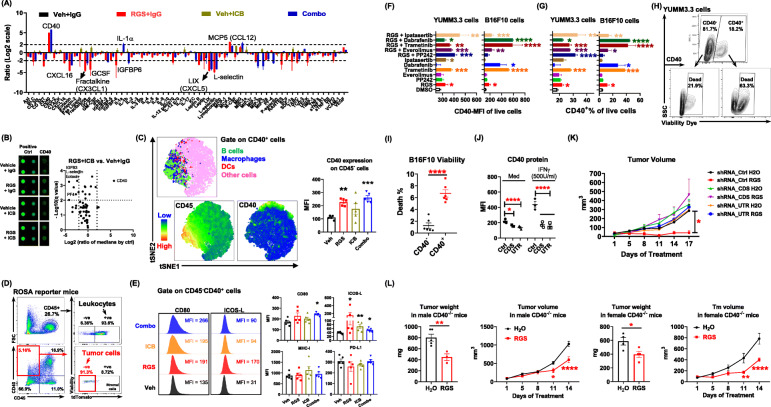
Rigosertib induces CD40 on melanoma cells and promotes the anti-tumor immunity. **a**, **b** Cytokine array of YUMM3.3 tumor lysate samples at day 17 post-treatment. **c** Live cells were concatenated after downsampling to ~ 20,000 events for t-SNE analysis through flow cytometry. **d** Flow cytometric analysis of YUMM3.3 tumors in ROSA reporter mice at day 14 post RGS treatment. **e** Mean fluorescence intensity (MFI) on the CD45^−^CD40^+^ cells isolated from day 17 YUMM3.3 tumors in C57BL/6 mice. **f**, **g** Melanoma cells were treated with indicated drugs for 48 h and CD40 expression was detected by flow cytometry. **h**, **i** Viability of YUMM3.3 and B16F10 cells was examined by flow cytometry. **j** Flow cytometric analysis of CD40 expression in response to treatment with IFNγ (500 U/ml, 48 h) in different clones of YUMM3.3 cells where shRNAs targeted coding sequence (CDS), 3″ untranslated region (UTR), or random sequences (Ctrl). **k** Tumor volume of CD40 knockdown clones in C57BL/6 mice was determined (*n* ≥ 4 mice per group). **l** Tumor weight and tumor volume of YUMM3.3 cells growing in C57BL/6-CD40 knockout male and female mice treated with RGS (300 mg/kg). **a**-**e** data were replicates from one experiment (*n* = 5 ~ 10 per group). **f**-**l** pooled data obtained from at least two different experiments (*n* = 3 ~ 6) are shown

Next, we profiled the immune effector cells in the TME. Within the lymphoid lineage, tSNE analysis showed that RGS induced a dose-dependent induction of T cells and NK cells while it reduced B cells in tumors (Fig. 3 a). We found that RGS increased not only the frequency of CD4^+^ T helper (Th) and CD8^+^ cytotoxic T cells (Tc) in the TME (Fig. 3 b, c), but also their activation, as reflected by elevated CD69 expression (Fig. 3 d). The percentage of tumor-infiltrating T cells was significantly and negatively correlated with the tumor burden (Spearman *p* < 0.0001, r = − 0.7924) (Fig. 3 e), suggesting that RGS-induced T cell responses play an important role in tumor control. The increased T cell frequency was further confirmed by IHC staining of the tumor sections (Fig. 3 f) and TDLNs (Suppl.Fig. 4). While the CD3^+^ and CD8^+^ tumor infiltrate was increased in the RGS-treated tumors, there was no significant alteration in the level of CD4^+^FoxP3^+^ T regulatory cells (Tregs) or angiogenesis (CD31) (Fig. 3 f). Notably, the percentages and activity of NK cells were increased in the RGS-treated tumors (Fig. 3 g). While there was an induction of DC, T cell, and NK cell responses within the RGS-treated tumors, we observed a 20% decrease of total CD11b^+^ myeloid lineage populations (Fig. 3 h, i). Among the specific myeloid populations, F4/80^+^ macrophages, especially the CD206^+^ M2-like macrophages, decreased most prominently in a dose-dependent manner in response to RGS treatment. While the level of MHCII^+^ M1-like macrophages did not correlate with the tumor burden, there was a significant and direct correlation between the decrease in CD206^+^ M2-like macrophages and tumor burden (Spearman *p* < 0.0001, r = 0.6400). To determine whether Th and Tc cells are required for the anti-melanoma activity of RGS, we conducted depletion experiments of CD4 and/or CD8 cells (Fig. 3 j). Interestingly, depletion of CD8 cells alone, or depletion of both CD8 and CD4 cells, but not depletion of CD4 cells alone, significantly abrogated tumor growth inhibition by RGS treatment (Fig. 3 k). These results suggest that CD8 cells are the main population responsible and necessary for RGS anti-tumor activity. We further identified that RGS-induced caspase-dependent cell death is important to induce the anti-tumor responses in vivo since the pan-caspase inhibitor Z-VAD treatment blocked RGS-induced tumor growth inhibition and reduced CD3^+^ T cell infiltration (Suppl.Fig. 5).

**Fig. 6:**
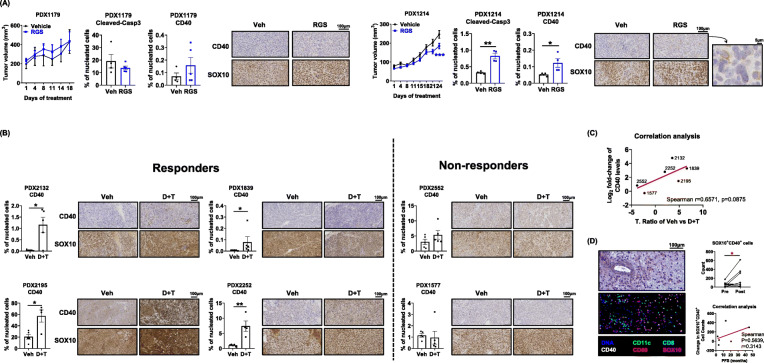
RGS and BRAF/MEK inhibitors induce CD40 expression on melanoma cells and tumors of patients responsive to RAS/RAF/MEK pathway inhibition. **a** NRAS^mut^ melanoma PDX 1179 and PDX1214 tumor growth over time in NSG mice with IHC staining of cleaved-caspase3, CD40 and SOX10 in from mice treated with the vehicle or 300 mg/kg rigosertib (RGS). 20X and 40X images and the scale marker are shown. **b** IHC staining of CD40 and SOX10 in BRAF^mut^ melanoma PDX tumors from mice treated with the vehicle or Dabrafenib + Trametinib (D + T). **c** Correlation analysis of T. Ratio and CD40 fold-change post treatment in PDXs shown in **b**. The T. Ratio, obtained from the statistical analysis of treatment difference comparisons of the tumor growth rate based on the tumor volume, for each PDX treatment comparison. **d** H&E and multiplex sequential IHC analysis of hematoxylin, CD40, CD80, CD11c, CD8 and SOX10 of patient melanoma sections. Cell number of SOX10^+^CD40^+^ cells was identified of 11 paired tumor sections pre- and post-BRAF inhibitor treatment (paired t-test). Pooled data (*n* = 4 ~ 11) from one experiment are shown

The safety profile of RGS in tumor-free (Suppl.Fig. 6A,B) and tumor-bearing (Suppl.Fig. 6C) mice was examined to evaluate the safety of RGS as a therapy. RGS treatment did not induce severe toxicity in mice, as there was no significant loss of body weight or increase in the levels of liver enzymes alanine aminotransferase (ALT) and aspartate aminotransferase (AST) in the serum of RGS treated as compared to the vehicle- treated mice. Given that in vitro treatment with RGS inhibited the mitosis/proliferation and promoted ICD of melanoma cells (Fig. 1 b, e & Suppl.Fig. 1), RGS’s impact on the cellular turnover of normal tissues (Suppl.Fig. 6B) was studied. No alteration of Ki67 levels was observed in the intestine, spleen, and skin tissue after 15 days of RGS treatment.

### Rigosertib improves responses of αPD-1/αCTLA-4 therapies

Since RGS treatment elicits an anti-tumor immune activation profile, we postulated that the addition of RGS to αPD-1/αCTLA-4 would improve therapeutic outcomes of melanoma tumors with poor response to ICB. The B16F10 model is reported to be highly aggressive and non-responsive to ICB therapies (such as αPD-1, αPD-L1, αCTLA-4 or a combination of αCTLA-4 with either αPD-1 or αPD-L1) [41], which makes it ideal to model clinical responses of melanomas that are either non-responsive to, or have progressed on, immune checkpoint inhibitors. Therefore, we pre-treated BRAF^WT^ /NRAS^WT^ B16F10 tumors with 3 doses of αPD-1 to validate the ICB non-responsive phenotype and then administrated either vehicle + IgG control, RGS + IgG, vehicle + αPD-1/αCTLA-4, RGS + αPD-1/αCTLA-4, or dabrafenib (BRAF inhibitor) + trametinib (MEK inhibitor) + αPD-1/αCTLA-4 as a control of clinically used combinatorial therapy (Fig. 4a). As expected, αPD-1/αCTLA-4 alone or in combination with dabrafenib + trametinib did not exhibit a beneficial effect on mouse survival (Fig. 4 b) or tumor growth inhibition (Fig. 4 c, d). In contrast, the combination of RGS plus αPD-1/αCTLA-4 improved mouse median survival to 22.5 days compared to 11 days in the vehicle + IgG control or monotherapy groups (Fig. 4 b) and resulted in ~ 70% inhibition of tumor growth (Fig. 4 c, d). The effects of RGS plus αPD-1/αCTLA-4 treatments were synergistic, based on a plot of interaction effect on tumor growth rate estimated by the mixed-effect model (Fig. 4 e). Detailed immune profiling of tumor-infiltrating leukocytes through tSNE analysis showed an improved T-cell response, especially CD8^+^ Tc cells, in tumors treated with either RGS monotherapy or with RGS in combination with αPD-1/αCTLA-4 (Fig. 4 f). The frequency of T cells within the TME increased from 2.6 ± 0.5% in the control group to 6.3 ± 1.5% with RGS treatment alone, and reached to 10.7 ± 1.5% in the RGS plus αPD-1/αCTLA-4 treatment group (Fig. 4 f).

**Fig. 7:**
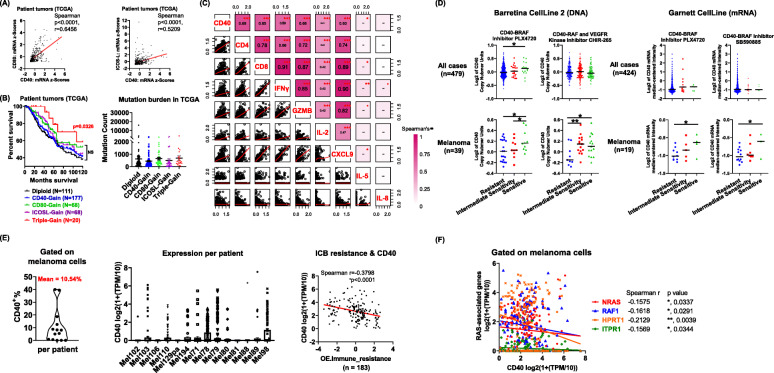
Correlations between CD40 expression in human melanoma and response to RAF inhibitors and ICB. **a** Correlations of CD40 gene expression with CD80 and ICOS-L expression from melanoma tumors in the TCGA dataset (*n* = 480) in cBioPortal. **b** Overall survival in reference to CD40, CD80 and ICOSL copy number and mutation burden in melanoma patients in TCGA-Melanoma dataset (*n* = 358). **c** Correlation matrix with Spearman’s rank correlation coefficients after data transformation analysis of CD40, CD4, CD8, IFNγ, GzmB, IL-2, CXCL9, IL-5 and IL-8 (y = (log(x-min(x) + 1))). **d** Drug sensitivity analysis of Barretina CellLine 2 (DNA) and Garnett CellLine (mRNA) human cancer cell line datasets in Oncomine database. **e, f** Analysis of gene expression levels and their correlation to ICB resistance. Gene expression in defined melanoma cells (*n* = 1883) were downloaded and analyzed based on single cell RNA-Seq (https://singlecell.broadinstitute.org/)

We next validated these results using BRAF^mut^ YUMM3.3 model. While αPD-1/αCTLA-4 therapy alone exhibited marginal growth inhibition of the YUMM3.3 tumors after 17 days of treatment, the addition of RGS to the αPD-1/αCTLA-4 regimen resulted in a significantly greater reduction in tumor volume (70%) (Fig. 4 g) as compared to RGS alone. While > 90% of tumors receiving either monotherapy (RGS or ICB) exhibited a rapid growth at 11 days post-treatment compared to baseline size, tumors under the combinatorial treatment grew a bit more slowly the first 11 days after treatment initiation (Fig. 4 h). Interestingly, there was a significant increase in Ki67^+^ cells in the tumors receiving either RGS or αPD-1/αCTLA-4 or both (Fig. 4 i). Flow cytometric analysis was performed to identify specific cell populations with high Ki67 expression. Analysis of the Ki67^+^ cells revealed that only 2.37% of the CD45^−^ cells, which includes tumor cells, were Ki67^+^ (Fig. 4 j). In sharp contrast, > 30% of CD45^+^CD3^+^ T cells were Ki67^+^, indicating that these T cells are proliferating in melanoma tumors. Notably, both RGS monotherapy and combinatorial treatments of RGS + αPD-1/αCTLA-4 increased the ratio of proliferating Ki67^+^ T-cell to CD45^−^ cells (Fig. 4 k). Further subtyping analysis suggested that RGS and combined RGS + αPD-1/αCTLA-4 treatments specifically promoted proliferation of CD8^+^ Tc cells, but not CD4^+^ Th cells nor the FoxP3^+^ Tregs while attenuating the proliferation of CD45^−^ cells in the TME (Fig. 4 l). Consistent with the immune profile in the TME, increased frequency and activation of NK cells, CD4^+^ Th and CD8^+^ Tc cells were also identified in the TDLN of the combinatorial treatment group (Suppl.Fig. 7). Notably, while αPD-1/αCTLA-4 treated tumors did not exhibit an elevated effector cell response at day 17 after treatment initiation, there was a clear induction of NK and CD4^+^ Th cell activities, as well as CD8^+^ Tc cell frequency, in the TDLN. The RGS plus ICB treatment did not result in significant loss of mouse body weight and did not increase spleen weight or serum levels of liver enzymes ALT and AST, as compared to the vehicle group (Suppl.Fig. 8).

To explore the cytokine production [IFN-γ, TNF-α and granzyme B (GzmB)] and antigen (Ag)-specificity of treatment-induced CD8^+^ Tc responses in the tumor microenvironment, we conducted in vitro recall experiments using the tumor-infiltrating T-cell samples isolated from above YUMM3.3 and B16F10 tumors (Suppl.Fig. 9). PBMCs isolated from tumor-free C57BL/6 mice were used as a negative control for antigen specificity. The stimulation with αCD3/CD28 beads was used as a positive control for CD8^+^ Tc cell responses. The production of TNF-α in tumor-infiltrating Tc cells was not detected in any tumor samples. IFN-γ in tumor-infiltrating Tc cells from B16F10 tumors was not detected. As expected, the positive control of αCD3/CD28 beads stimulated IFN-γ production in > 80% of Tc cells isolated from the tumor-free peripheral blood samples (Ag-naive PBMC). The autologous antigen stimulation from parental YUMM3.3 tumor irradiated cells significantly induced IFN-γ production in ~ 20% of Tc cells isolated from tumors undergo RGS + ICB treatment, compared to either Ag-naive PBMC or Tc cells isolated from tumors that were treated with vehicle+IgG. Interestingly, αCD3/CD28 beads did not stimulate GzmB^+^ Tc cells in the PBMC samples (stimulated for 4 h at 37 °C and 4 °C overnight). This observation is consistent with the report that the induction of GzmB-containing cytotoxic granules in T cells using stimulation with αCD3/CD28 beads, required at least 3 days, occurred after several rounds of cell division, and required cell cycle progression [42]. Nevertheless, cells isolated from tumors treated with RGS + IgG exhibited an increase of GzmB^+^ Tc cells in both B16F10 and YUMM3.3 tumors. In the RGS + IgG group, there was a significant induction of GzmB^+^ Tc cells with stimulation of irradiated YUMM3.3 cells compared to Ag-naive PBMC samples, which is comparable to the level in the positive control of αCD3/CD28 beads, suggesting that the RGS-induced Tc cell response is at least partially antigen-specific.

The programmed death-ligand 1 (PD-L1)/PD-L2/PD-1 axis delivers inhibitory signals that function as a brake for immune responses in cancer [43]. Recently, PD-L1^+^ B cells were identified to be a feature of regulatory B cells (Bregs) that are critical regulators of anti-cancer immunity [44, 45]. We found that RGS and/or ICB (αPD-1/αCTLA-4) treatments did not alter B cell generation in the bone marrow (Suppl.Fig. 10A). Instead, there was a significant reduction of B cells in the tumor, TDLN, and spleen tissues under the RGS + ICB treatment. The reduction of tumor-infiltrating B cells was confirmed by IHC staining (Suppl.Fig. 10B). While all the tumor-infiltrating B cells were PD-L1^+^ Bregs, some of them were co-expressing PD-L2^+^ and belong to the antibody-producing follicular B cell (FOB) lineage (Suppl.Fig. 10C). Detailed profiling of the B cell subsets in the TME [46] suggested that while the RGS + ICB treatment attenuated the FOB population, it promoted the CD5^−^ B1b cells (Suppl.Fig. 10D,E). Though there is very limited functional characterization of how these B cell subsets may perform in tumor immunity in the literature, B1b cells were reported to confer T cell-independent long-lasting protective immunity against infections [47].

### Rigosertib induces CD40 on melanoma cells and fuels the anti-tumor immunity

In order to gain further insight into molecular mechanisms of anti-tumor activity of RGS and RGS + ICB combined treatments, tumor cytokines in drug-treated versus vehicle-treated tumor-bearing mice were examined using a cytokine array (Fig. 5 a, b). We observed that CD40 was increased ~ 30-fold in tumors from mice that received either RGS monotherapy or the RGS + αPD-1/αCTLA-4 combined treatment as compared to control vehicle-treated mice. While both antigen-presenting cells (APCs, including DCs, macrophages and B cells) and melanoma cells were reported to express CD40 [26, 27], subsequent tSNE analysis by flow cytometry showed that in our experimental setting RGS treatment-induced CD40 expression on CD45^−^ cells but not on APCs (Fig. 5 c). To identify the origin of these CD40^+^CD45^−^ cells, we inoculated YUMM3.3 melanoma cells into mice harboring the tdTomato, which was inserted into the *Gt (ROSA)26Sor* locus (ROSA mice) so that every single cell originating from these mice would exhibit tdTomato^+^ in flow cytometric analysis [48]. As expected, ~ 93.6% of the CD45^+^ leukocytes were tdTomato^+^, confirming that they originate from ROSA mice (Fig. 5 d). We observed that ~ 91.3% of the RGS-induced CD40^+^CD45^−^ cells did not express tdTomato (tdTomato^−^), suggesting that they were tumor cells that were injected into mice to generate tumors rather than the endogenous stromal cells from ROSA mice. Of interest, these CD40^+^CD45^−^ cells in RGS-treated mice exhibited increased expression of co-stimulatory molecules CD80 and ICOS-L, with a baseline level of MHC-I and unaltered PD-L1 expression (Fig. 5 e). Costimulatory molecules, such as CD40, CD80 and inducible T-cell co-stimulator ligand (ICOS-L), all provide signals for activating cell-mediated immune responses [49, 50], further indicating that RGS treatment is inducing an immune response.

As a RAS mimetic, RGS has the potential to block oncogenic RAS-RAF-MEK-ERK and/or PI3K-AKT-mTOR signaling pathways [15–17]. To explore whether RGS could directly induce CD40 expression in melanoma cells, and if so, which RAS-mediated pathway(s) may be responsible for the phenotype, we evaluated the impact of serval pathway specific inhibitors on CD40 plasma membrane expression in melanoma cells in vitro, including mTOR kinase inhibitor PP242 (5 μM), mTORC1 inhibitor Everolimus (10 nM), MEK inhibitor Trametinib (0.1 μM), BRAF inhibitor Dabrafenib (1 μM), AKT inhibitor Ipatasertib (10 μM), and/or RGS (0.1 μM). We observed that RGS directly induced CD40 intensity (Fig. 5 f) and the frequency of CD40-membrane positive cells (Fig. 5 g) in B16F10 and YUMM3.3 cells. While mTOR inhibition (PP242 and Everolimus) did not promote CD40 expression on melanoma cells, suppression of either MEK or AKT pathways, induced CD40 expression in BRAF^mut^ YUMM3.3 cells. The inhibition of MEK or BRAF in BRAF^WT^ B16F10 cells resulted in a ~ 10-fold increase of CD40 expression. Notably, when RGS was combined with any of these RAS pathway inhibitors, the induction of CD40 expression in melanoma cells was further increased, suggesting pathways downstream of RAS may cooperate to restrain baseline CD40 expression in melanoma cells. It has been reported that CD40 signaling induces malignant cell death in many cancer types, including renal cell carcinoma [29], urothelial cell carcinoma [30], ovarian carcinoma [31, 32], cervical carcinoma [33] and bladder carcinoma [34]. Here, we identified that the death rate of CD40^+^ melanoma cells was 3-fold higher than in CD40^−^ melanoma cells for both RGS-treated YUMM3.3 (Fig. 5 h) and B16F10 models (Fig. 5 i).

To determine whether induction of CD40 expression was required for melanoma tumor cell response to RGS, we next knocked down the expression of CD40 in YUMM3.3 melanoma cells (CD40KD) by targeting either the coding sequence (CDS) or the 3′ untranslated region (UTR) to achieve ~ 60% knockdown of CD40 mRNA (Suppl.Fig. 11) and protein expression (Fig. 5 j). The CD40KD melanoma cells were resistant to IFNγ-mediated induction of CD40 expression (Fig. 5 j). Furthermore, while shRNA control vector transfection responded to RGS treatment with reduced YUMM3.3 tumor growth in vivo, tumors with attenuated CD40 expression (shRNA_CDS and shRNA_UTR) were resistant to RGS-mediated inhibition of tumor growth (Fig. 5 k). These data illustrate a requirement of melanoma cell expression of CD40 for RGS-mediated tumor growth control. To evaluate the importance of host-derived CD40 in RGS-induced responses, we injected YUMM3.3 cells into CD40 knockout (CD40KO) mice (Fig. 5 l). Notably, YUMM3.3 tumors responded to RGS treatment in CD40KO mice, however the response was not as prominent as in CD40 wild-type mice (30 ~ 45% growth inhibition post-two-weeks of treatment vs. 50%, respectively). These results suggest that melanoma cell-intrinsic CD40, rather than host-derived CD40, plays a major role in the RGS-mediated inhibition of tumor growth. In addition, we explored the CD40 ligand (CD40L) expression on T cells in the tumor microenvironment (Suppl.Fig. 12) and found that ~ 50% and ~ 10% of CD4^+^ Th cells express CD40L in the YUMM3.3 and B16F10 tumors, respectively. Notably, RGS + ICB significantly induced the frequency of CD40L^+^ Th cells in the tumor microenvironment. Compared to the Th cells, CD8^+^ Tc cells exhibited a more abundant CD40L expression with Tc cells from YUMM 3.3 cells being ~ 90% CD40L^+^ and from B16F10 tumors being ~ 40% CD40L^+^. The availability of CD40L on tumor-infiltrating T cells support our proposed model of CD40-dependent and Tc-dependent immunogenic cell death of tumor cells. Of note, CD4^+^ Th cells from tumors treated with RGS exhibited a 50% increase in CD40L expression, while CD4^+^ Th cells from RGS + ICB treated tumors exhibited only about a 15% increase in CD40L expression in YUMM3.3 tumors. In contrast, B16F10 tumors treated with RGS + ICB exhibited the greatest increase in CD40L expression. Collectively, our data indicate that the inhibition of RAS/RAF/PI3K-mediated pathways, as observed with RGS treatment, directly induce CD40 expression in melanoma cells to trigger immunogenic cell death, which is associated with increased expression of co-stimulatory signals (e.g., CD40, CD80, and ICOS-L) for mediating anti-tumor responses.

### Rigosertib and BRAF/MEK inhibitors induce CD40 expression in responsive patient melanoma cells in vivo

To determine whether these findings regarding induction of CD40 expression observed in mouse melanoma models are recapitulated in human melanoma, we evaluated the effects of RGS on the growth of two human melanoma PDX tumors previously established and characterized in our laboratory [51]. PDX1179 had an NRAS^Q61H^ mutation and PDX1214 had NRAS^Q61L^ and TP53 mutations (Suppl.Table 1). When these PDX tumors were implanted into NGS mice and evaluated for response to RGS as compared to vehicle, PDX1179 was completely resistant to RGS treatment, while PDX1214 exhibited ~ 25% growth inhibition over 3 weeks of treatment (Fig. 6 a). Notably, we observed a significant increase in the levels of cleaved-caspase3 and CD40 in PDX1214, but not in PDX1179. As expected, the major cellular population within these PDX tumors was SOX10^+^ melanoma cells. Notably, in PDX1214 the SOX10 expression pattern matched well with the CD40 expression pattern based upon IHC staining, even though there was only minimal inhibition of tumor growth. Since NGS mice lack a functional adaptive immune system, it is expected that any anti-tumor effects of RGS would be largely driven by the direct effect on PDX growth. Indeed, similar findings were observed of murine melanoma tumors (YUMM3.3 and B16F10) grown in athymic mice that do not have mature T cells, which were completely resistant to RGS (Suppl.Fig. 13), suggesting that the adaptive immune system is indispensable for the RGS-induced anti-tumor effects.

Since dabrafenib and trametinib also induce CD40 expression on melanoma tumor cells, we further examined response to this therapeutic combination (30 mg/kg dabrafenib + 1 mg/kg trametinib, D + T) in mice harboring mutated BRAF^V600^ patient-derived tumors (Fig. 6 b). Based on our published work [51], PDXs can be stratified into 2 distinct categories based of their sensitivity to D + T: responders (PDX2132, PDX1839, PDX2195 and PDX2252) and non-responders (PDX2552 and PDX1577). At the baseline level, CD40 expression varied among these PDXs ranging from 0.1 to 20% of all nucleated cells. Analysis of CD40 and SOX10 expression using IHC staining in BRAF^V600^ PDXs consistently showed a significant 3- to 15-fold induction of CD40 expression in all 4 responsive tumors compared to the respective vehicle control treated tumors. This was not the case in non-responsive tumors. To facilitate comparisons among PDX tumors and the responses to therapy, T-ratios for treatment group comparisons across these BRAF^V600^ PDX tumors were generated (Fig. 6 c). The T-ratio is a ratio of the difference in the slope of tumor growth between control and a treatment group relative to its SE, calculated by estimated marginal means based on the mixed-effect model. We found a clear trend towards a positive correlation between the T-ratios of drug responsiveness and the fold increase of CD40 levels post D + T treatment (Spearman r = 0.6571, *p* = 0.0875).

We next conducted 6-color (hematoxylin, CD40, CD80, CD11c, CD8 and SOX10) multiplex IHC staining on the section of tumors from melanoma patients treated with BRAF inhibitor (Fig. 6 d). As expected, we observed CD40^+^SOX10^+^ melanoma cells in the TME of all 11 post-treatment samples and in 10 of 11 pretreatment samples. The CD40^+^SOX10^+^ cells in each tumor biopsy ranged from 2 to 616 counts. The number of CD40^+^SOX10^+^ melanoma cells increased to 103 post BRAF treatment in the one tumor that did not have CD40^+^SOX10^+^ cells prior to BRAF inhibitor treatment. Notably, we observed a significant induction of CD40^+^SOX10^+^ cells in the tumors when pre-BRAF inhibitor treatment (mean counts = 57) was compared to post-BRAF inhibitor treatment tissue (mean counts = 154). Although we did not detect a statistically significant correlation between the change in the number of CD40^+^SOX10^+^ cells in the TME post-treatment and the progression free survival (PFS) due to the small sample size and complications of prior treatments, we observed a clear trend for a positive correlation for D + T induction of CD40^+^SOX10^+^ cells for the 6 melanoma patients studied who had not undergone any treatment prior to BRAF/MEK inhibitor therapy (Spearman r = 0.3143). Together, these data suggest that CD40 is induced in tumor cells of melanoma patients responding to the therapies targeting RAS/RAF/MEK pathway.

### Correlation of CD40 in survival and response to therapies in melanoma

We next investigated whether CD40, CD80, and ICOS-L expression correlates with the outcome and therapeutic response in melanoma patients. By querying The Cancer Genome Atlas (TCGA, *N* = 480) patient datasets of melanoma samples, we found that the CD40 expression was significantly correlated with CD80 (Spearman *p* < 0.0001, r = 0.6456) and ICOS-L (Spearman *p* < 0.0001, r = 0.5209) in tumors from melanoma patients (Fig. 7 a). Given that RGS treatment in mice increased CD40, CD80, and ICOS-L expression levels on tumor cells, and this was associated with a better therapeutic outcome, we questioned whether the levels of CD40/CD80/ICOS-L have any prognostic value in melanoma patients. While the loss of CD40 copy number is very rare in melanoma patients (~ 1.25%, TCGA), ~ 50% of melanoma patients exhibit CD40 copy number gains (Fig. 7 b) and thus may be prone to exhibit enhanced inducible expression of CD40. Though the increased copy number of either CD40, CD80 or ICOS-L alone did not correlate with any changes in patient overall survival (OS), the triple-gain group of patients (*n* = 20) who exhibited increased copy number of CD40, CD80, and ICOS-L, exhibited a significantly better 10-year OS (*p* = 0.0326) (Fig. 7 b). This triple-gain group of patients did not exhibit a higher mutational burden (Fig. 7 b). Consistently, a correlation matrix profiling of gene mRNA expression in TCGA collection of melanoma tumors showed that CD40 expression significantly and positively correlates with CD4 and CD8 infiltrates, as well as with type 1 cytokine responses based on IFNγ and GzmB expression patterns (Fig. 7 c). In addition, increased T cell responses may be a result of elevated T cell recruitment (based on association with chemokine CXCL9 implicated in T cell trafficking) and to a lesser extent, T cell proliferation (IL-2). Notably, type 2 cytokine responses (IL-5) and neutrophil responses (CXCL8/IL-8) were not altered by the changes of CD40 expression, suggesting that CD40 expression specifically correlates to the T-cell-dependent antitumor immunity in human melanoma tumors.

Given that RGS interferes with RAS-RAF activation and triggers CD40-dependent anti-tumor effects in melanoma, we questioned whether the CD40 level is prognostic of response to previously used effective therapy, such as RAF inhibitor treatment. Thus, we further conducted drug sensitivity analysis of Barretina Cell Line 2 (DNA) and Garnett Cell Line (mRNA) datasets that are available in the Oncomine database (Fig. 7 d & Suppl.Fig. 14). While the CD40 level does not correlate with the drug sensitivity to RAF inhibitors for all cancer types, human melanoma cells with increased CD40 at either copy number or mRNA levels are significantly more sensitive to the RAF inhibitor treatments. In contrast, CD80 and ICOSL are not correlated to the sensitivity of melanoma cells to the BRAF inhibitors (Suppl.Fig. 15).

Given the immune-modulatory role of CD40 to promote DC and T-cell responses, we next questioned whether expression of CD40 would correlate with response to ICB immune therapy in melanoma. To do so, we analyzed the bulk tumor RNA-Seq results of the two largest available melanoma datasets with both transcriptomics and ICB immunotherapy response [52, 53]. We found that CD40 expression is significantly higher in tumors from melanoma patients that respond to ICB compared to the non-responsive patients (Suppl.Fig. 16). To further dissect the prognostic value of melanoma cell-intrinsic CD40 to ICB, we retrieved and analyzed the single cell (sc) RNA-Seq dataset of melanoma patients from Single Cell Portal (Broad Institute) [54]. This dataset contains gene expression and ICB response profiles from 14 melanoma patients from whom 1883 melanoma cells were sc-sequenced (Fig. 7 e). We determined that the mean frequency of CD40 positive melanoma cells in these patients is 10.54%. There is a wide range of CD40 expression per patient and for 3 out of 14 patients there was no detectable CD40 expression in the melanoma cells. Notably, CD40 expression in a patient’s melanoma cells significantly and negatively correlates with the resistance level to ICB immunotherapy. To explore whether *RAS* and *RAS*- mediator expression levels correlate with *CD40* expression in a patient’s melanoma cells, we further screened the mRNA expression profile of genes in the N-RAS regulation pathway microarray (Bio-Rad) (Fig. 7 f). Among the 20 genes examined, 4 of them (including *NRAS*, *RAF1*, *HPRT1* and *ITPR1*) exhibited a significantly negative correlation with the *CD40* expression level in melanoma cells, suggesting a potential negative regulatory role for RAS signaling in *CD40* mRNA expression in melanoma cells from patient tumors. Together, these data suggest that melanoma cell-intrinsic CD40 correlates with response to ICB and response to inhibitors of the RAS-mediated pathways, including RGS, in melanoma patients.

## Discussion

In this preclinical study, we first determined that RGS treatment reduces melanoma tumor growth and enhances response to ICB therapy. The combinatorial therapy of RGS + ICB exhibited stronger anti-tumor effects compared to ICB monotherapy and favored an inflammatory CD8 Tc cell response in the TME and TDLN (Fig. 4). We determined that the optimal in vivo dose of clinical-grade RGS [55] to treat melanoma tumors in mice by oral gavage is 300 mg/kg/day (dosed 5 days a week), which was well tolerated. This is consistent with the reported safety profile of RGS in clinical trials. Notably, in a recent phase II/III trial of metastatic pancreatic cancer [24] and phase III trial of MDS [21], patients received RGS 1800 mg/m^2^ via 2 h continuous IV infusions given twice weekly for 3 weeks of a four-week cycle or 1800 mg per 24 h via 72 h continuous intravenous infusion administered every other week, which is equivalent to ~ 600 mg/kg and ~ 370 mg/kg in mouse, respectively [56]. An oral formulation of RGS (dose escalation to maximal dosing 560 mg BID on days 1 to 14 of a 21-day cycle or 1 to 21 of a 28-day cycle respectively) is being studied in phase I/IIa trials in head and neck squamous cell carcinoma (NCT01807546) and non-small cell lung cancer (NCT04263090). Given the promising safety and therapeutic profile of RGS combined with our data showing RGS can enhance response to ICB therapy in melanoma, our data indicate that a combination of RGS + ICB therapy warrants a clinical study in melanoma patients. Notably, tumor growth rate upon treatment with RGS alone was comparable to that of ICB, suggesting that treatment alone may be effective in slowing tumor growth for melanoma patients with primary or secondary resistance to ICB treatment.

While RGS reduced viability in vitro in human and murine melanoma cells independent of their mutational status (Fig. 1), we observed that RGS exhibited little inhibition of tumor growth in immunodeficient mice (Fig. 6 & Suppl.Fig. 13). These data suggest not only that tumor cell-intrinsic effects of RGS are insufficient to restrain melanoma tumor growth in vivo, but also reinforce the key role of RGS-activation of anti-tumor immunity in its therapeutic role. Of note, dying cells are known to elicit a wound healing response in neighboring cells, so-called apoptosis-induced proliferation (AiP), that promotes proliferation of the surrounding cells [57]. Consistently, we observed a simultaneous increase of apoptotic (cleaved-caspase3) and mitotic (PLK1 and FOXM1) markers at 24 h post RGS treatment in melanoma cells (Fig. 1 c &Suppl.Fig. 1). Based on the work in several model organisms, caspases drive AiP through ROS generation [58]. We observed RGS-induced caspases and ROS in melanoma cells. While a direct role of AiP has not been demonstrated here, AiP induction is a plausible mechanism for the RGS resistance we observed in the immunodeficient mice. Future work will need to address this question.

The work described here illustrates that RGS not only has direct melanoma cell killing effects, but it also induces immunogenic cell death to facilitate response to ICB immunotherapy, which is associated with elevated DC and Tc cell responses (Figs. 2, 3, 4). Notably, the RGS induced CD40 upregulation on melanoma cells, which is associated with cell death and tumor antigen release, indicating that CD40 was required for the induction of anti-tumor immune responses (Fig. 5). These data are consistent with the recent reports that CD40-mediated signaling in tumor cells induces tumor cell death, while in normal cells, CD40 signaling is cytoprotective [32, 59]. Of note, CD40 agonists have been combined with chemotherapy to enhance the capacity for T cell stimulation, especially in solid malignancies. However, no clinical benefit was observed with delivering chemotherapy prior to a CD40 agonist [60, 61]. In contrast, preclinical models have shown that CD40 agonist treatment enhanced sensitivity to gemcitabine chemotherapy via the up-regulation of proinflammatory IFNγ responses [61], which supports a role for delivery of CD40 agonists in sensitizing tumors for subsequent sequential therapies. Currently, several clinical trials are ongoing using the CD40 agonists APX005M: 1) in combination with nivolumab and cabiralizumab (CSF1R antagonist) in patients with advanced melanoma, non-small cell lung cancer or renal cell carcinoma whose disease has progressed on α-PD-1/PD-L1 therapy (NCT0302330); 2) in combination with a personal cancer vaccine (neo-pv-01) or ipilimumab with nivolumab in patients with advanced melanoma (NCT03597282); 3) in combination with nivolumab in patients with non-small cell lung cancer or metastatic melanoma (NCT03123783). It will be interesting to see how systemic activation of CD40 affects disease in these trials and to sort out mechanisms by which any observed effects occur.

Indeed, intratumoral CD40 activation in combination with ICB induces T cell-mediated eradication of melanoma in animal models [62]. However, safety concerns (e.g., thromboembolic events and cytokine storm release) limited the clinical development of some CD40 agonists early on [63]. Notably, when applying agonistic CD40 antibodies locally instead of systemically, adverse events were shown to be diminished [64, 65]. Our data show that CD40 expression is prognostic for therapeutic response to RGS, RAF inhibitor and ICB treatments in human melanoma cells (Fig. 7). The induction of CD40, as well as CD80 and ICOS-L, in RGS-treated melanoma tumors, may form a positive feedback loop to further sensitize tumors to RGS treatment. We identified a gain of CD40 copy number (CD40-gain) in 177 out of 358 TCGA-melanoma patients (Fig. 7 b). While we showed that inhibition of RAS-mediated pathways would induce CD40 on melanoma cells, based on prior work showing poor induction of CD40 expression in CD40 low cells [27, 28], induction of CD40 expression by RGS or other therapies may be more effective for those tumors that are CD40 diploid, or with increased CD40 copy number (CD40-gain). Recently, a systematic pan-tumor analyses investigated ICB-response biomarkers in a cohort of over 1000 ICB-treated patients from 12 individual studies, consisting of 7 tumor types, namely urothelial, head and neck, renal, colorectal, breast, non-small cell lung cancer and melanoma, treated with 3 distinct ICB-classes (α-CTLA-4, PD-1, or PD-L1) [66]. This meta-analysis study revealed the importance of both tumor-cell-intrinsic (e.g., tumor mutational burden and PD-L1 expression) and tumor-cell-extrinsic (e.g., CXCL9, CD8A, CXCL13, CCR5 and T cell inflamed gene expression signature) factors underpinning ICB sensitization. While ICB may reinvigorate T cells by releasing the brake on T cell activation, many of these ICB-response factors may further modulate the response and the final therapeutic outcome. In our preclinical melanoma models, both B16F10 and YUMM3.3 tumors do not respond to ICB monotherapy (Fig. 4). The targeted therapy of rigosertib increased response to ICB and converts the “non-responders” into “responders”.

There are limitations to our study. For instance, the analysis of protein markers and immune profiling in melanoma tumors should be interpreted with caution; at the end point of tumor tissue collection, some of the tumors were large with regions of necrosis (particularly in the control group). This and other potential variables that are not directly related to therapy may influence the microenvironment within the tumors and increase variability among tumors. Another limitation is that clinical trials to test the therapeutic effects of RGS in melanoma have not occurred at this time. Due to the small sample size of paired BRAF inhibitor samples from the BRAF inhibitor clinical trial, we could only identify a trend toward a correlation between CD40^+^ melanoma and patient survival, which warrants future trials to validate the role of CD40^+^ melanoma cells in treatment responses. Moreover, there is recent controversy around the microtubule-destabilizing activity of RGS preparations as to whether it is due to a contaminant or endogenous activity of RGS [67, 68]. Of note, several clinical trials are investigating the effectiveness of combining microtubule inhibitors with ICB therapy in melanoma (NCT01827111, NCT00796991 and NCT02617849). Here we are predominately studying the effect of RGS on ICD and downstream immune responses, so regardless of whether RGS is inducing ICD by directly inhibiting the RAS/RAF/MAPK/PI3K pathway or microtubules, the results are highly significant.

One particularly interesting finding from our study is the unexpected upregulation of CD40 in the melanoma tumor cells following inactivation of RAS-mediated signaling by RGS or dabrafenib plus trametinib treatment. Notably, RGS does not inhibit kinase activities of A-Raf, B-Raf, or C-Raf when added in vitro to purified Raf proteins [18, 38]. However, the level of Raf dimerization was dramatically reduced in HeLa cells with EGF stimulation [38]. It is well established that the RAS/RAF/MEK pathway is essential for the proliferation, survival, and progression of most tumor types. We postulated initially that suppression of the RAS/RAF/MEK and PI3K pathways might promote immune surveillance through increased antigen release from dying tumor cells. However, we clearly cannot rule out the possibility that CD40 upregulation itself is the key event associated with the RGS-initiated suppression of tumor growth. In fact, under otherwise identical conditions, response to RGS was reversed by (i) suppression of CD40 expression in the tumor cells by shRNA, and (ii) depletion of CD8^+^ Tc cells. Both results are consistent with the hypothesis that RGS markedly enhanced immune surveillance of the tumor through CD40-upregulation. Given the importance of RAS/RAF/PI3K pathway activation across a very broad spectrum of human cancer types, it is worth considering the possibility that, in addition to its effects on cell proliferation/survival, RAS/RAF/PI3K-pathway activity also directly masks immune surveillance during the early stages of tumor progression.

## Conclusions

This study provides proof of concept that the inhibition of RAS/RAF/PI3K-pathway induces CD40 upregulation and immunogenic cell death in melanoma cells, which sensitizes them to ICB therapy. The observations here point to the potential usefulness in examining CD40 expression as a possible prognostic marker for response to ICB therapy as well as a need for new clinical trials combining RGS with ICB in metastatic melanoma patients.

## Materials and methods

### Study design

The aim of the study was to determine the effectiveness and mechanism of RGS as a monotherapy and in combination with ICB in melanoma. Cell viability and cell death assays through flow cytometry, western blotting and IHC staining supported the cytotoxic effect of RGS on melanoma cells, but not the normal melanocytes. Detailed immune profiling through RNA-Seq, flow cytometry and IHC staining of tumor and lymph node samples was used to determine the role of RGS in shaping the TME and the results were confirmed with CD8 cell depletion. Cytokine array, cell lineage tracking by flow cytometry and shRNA knockdown were used to identify the requirement of CD40^+^ melanoma cells in RGS-induced immune responses. Patient-derived xenograft models and multiplex IHC analysis of melanoma tumor samples from BRAF inhibitor trial were used to validate the role of CD40^+^ melanoma in human. Lastly, we conducted human melanoma database analysis, including TCGA, Oncomine and Single Cell RNA-Seq Portal, to determine the prognostic value of melanoma-expressing CD40 in patient survival and response to RAF inhibition and ICB therapies.

### Cell lines

Human melanoma cell lines [A375; SkMel2; SkMel5; HS294T], the B16F10 murine melanoma cell line and the HEK293 cell line were purchased from the American Tissue Culture Collection (ATCC). Murine melanoma cell lines [YUMM 2.1; YUMM 3.3; YUMM 4.1; YUMM 5.2] were provided by Marcus Bosenberg (Yale University). The status of driver mutation genes in melanoma cell lines were listed in Suppl. Table 1. The genetics of the YUMM cell lines was verified by RNA-Seq. Human Mel-ST and murine Melan-A melanocytes were provided by Robert Weinberg (Massachusetts Institute of Technology) and Dorothea Bennett (St. George’s Hospital Medical School), respectively. All cell lines were free of mycoplasma contamination.

### Mouse tumor models

Animal studies were approved by the Vanderbilt Institutional Care and Animal Use Committee (IACUC) and were performed in accordance with Vanderbilt IACUC guidelines. All animals were housed under pathogen-free conditions at the Vanderbilt Animal Care Facility. C57BL/6 mice, athymic nude mice, CD40 knockout, and NSG mice were purchased from Jackson Labs. Tumor xenografts were established in 8–10 week-old male or female mice. For the in vivo melanoma model, mice received 3 × 10^5^ of tumor cells in 100 μl of serum-free DMEM medium by subcutaneous injection in the lower back. Mouse body weight was assessed once a week, and tumor measurements were taken twice a week with micro-calipers. Tumor volume was estimated as 0.5*length*width*width. Treatment began when tumors reached ~100mm^3^ volume on average or at day 10 post tumor cell inoculation and continued until tumors in the experiment exceeded 15 mm in diameter or became perforated.

### Patient material

All patient donors signed an approved informed consent before providing tissue samples. Patient samples were collected on a tissue-collection protocol approved by the Vanderbilt University IRB. Melanoma PDXs in NSG mice were established from melanoma tumors obtained from patients who were referred for surgical resection or biopsy and propagated, as described previously [51, 69]. The status of driver mutation genes in melanoma PDXs were listed in Suppl. Table 1. Paired advanced melanoma samples from 11 patients (pre- and post-treatment of BRAF inhibitors) were collected as part of NCT01205815 clinical trial (Suppl.Table 2).

### Therapeutic treatment regimens

Rigosertib (300 mg/kg, Onconova Therapeutics) was administered 5 days a week by oral gavage in a total volume of 100 μL. Immunotherapies or equivalent amounts of isotype control, including anti-mouse PD-1 (clone: RMP1–14), Rat IgG2a control (clone: 2A3), anti-mouse CTLA-4 (clone: 9H10) and polyclonal Syrian hamster IgG control, were administered intraperitoneally at 100 μg per mouse every 3 days for two weeks. For depletion of CD4^+^ and CD8^+^ cells, mice were injected intra-peritoneally with 200 μg of anti-mouse CD4 (clone: GK1.5), anti-mouse CD8α (clone: YTS 169.4), or the same amount of rat IgG2b control (clone: LTF-2) antibodies 3 days prior to the tumor cell injection and every 3 days thereafter for the entire experiment. All antibodies were purchased from BioXcell. For caspase inhibition, pan-caspase inhibitor Z-VAD (APExBIO Technology), or the same amount of DMSO control in PBS, were given intra-peritoneally daily at 2 mg/kg for 14 consecutive days. PP242 (SelleckChem, #S2218), Everolimus (SelleckChem, #S1120), Dabrafenib (NCI), Trametinib (NCI) and Ipatasertib (MedKoo, #205467) were reconstituted in dimethyl sulfoxide (DMSO) and used for in vitro culture.

### Western blot

RIPA buffer (Sigma-Aldrich) was used to prepare lysates and protein concentration was measured using Bradford Protein Assay reagent (BioRad). Whole-cell extracts were separated on 4–20% Mini-Protean TGX Gels (BioRad) and transferred using Trans-Blot Turbo RTA Transfer Kit (Nitrocellulose, BioRad). Primary antibodies were used at 1:500 dilution, except antibodies recognizing housekeeping protein HSP90, which were used at 1:2000. All primary antibodies were applied overnight at 4 °C. See Suppl. Table 3 for primary antibodies utilized in this study. Secondary anti-rabbit or anti-mouse antibodies were used at 1:5000 dilutions for 1–2 h at room temperature.

### Cell viability assays

The CellTiter-Blue® Cell Viability Assay was performed according to the manufacturer’s recommendations. The percentages of viable cells and IC50s were calculated and presented in relevance to untreated control (100% viable) using Prism 8.0.1 software (Graphpad). Crystal violet staining was performed with a commercial 1% solution (Sigma-Aldrich) on cells that were fixed with 100% methanol for 10 min. Images were captured using a EVOS XL Core Imaging System (Invitrogen).

### Flow cytometric analysis

Tumor tissues were minced on a programmable gentleMACS dissociator (Miltenyi Biotec) and digested with an enzyme solution of collagenase I (1,500 CDU, Worthington Biochemical Corp.), Dispase II (1 mg/mL, Worthington Biochemical Corp.) and DNase I (0.1 mg/mL, Worthington Biochemical Corp.). Digested tumors were passed through a 70-μm strainer to obtain a single-cell suspension. Minced samples of mouse spleens and lymphoid nodes (LN) were pressed through a 70-μm strainer using a syringe plunger to obtain a single-cell suspension. Bone marrow (BM) was flushed with 5% FBS RPMI, centrifuged, resuspended, and passed through a 70-μm strainer for single-cell suspension. Erythrocytes were lysed by incubation in an ammonium-chloride-potassium buffer for 5 min at room temperature. The detail of antibodies used (Suppl.Table 4), staining, and flow cytometry analyses protocols is according to our previously published methodology [70]. Briefly, cells were incubated with Ghost Dye Violet 510 (Tonbo Biosciences), an amine-reactive viability dye used to discriminate live/dead cells and washed with FACS buffer (PBS containing 1% v/v FBS). After blocking Fc receptors with anti-mouse CD16/CD32 mAb (BD Biosciences) in FACS buffer for 20 min, cells were incubated with target antibodies. After staining, cells were washed twice in FACS buffer and fixed with 300 μl/tube of fixation buffer (1% formalin in PBS). Data were collected using a BD LSR Fortessa flow cytometer and analyzed using FlowJo software (Version 10.5.3). For t-SNE analysis, live CD45^+^ leukocytes were concatenated after downsampling to 12,500 ~ 20,000 events for subsequent high-dimensional data analysis to normalize the contribution among samples under different treatments. Samples were then analyzed in parallel by t-SNE and manually gated leukocyte populations were overlaid onto the total t-SNE map using FlowJo 10.5.3. For cell culture samples, apoptosis was assessed using an Annexin V-FITC Apoptosis Detection Kit (eBioscience). Reactive oxygen species (ROS) production was determined using the MitoSOX™ Red reagent (Invitrogen). For intracellular flow cytometric analysis on the cytokine production and antigen specificity of tumor-infiltrating CD8^+^ Tc cells, we followed the protocol as described previously [71]. Frozen cells isolated from YUMM3.3 and B16F10 tumors (*n* = 3 per treatment group), containing 0.2 million T cells, were thawed and recovered in complete RPMI medium with 10^2^ U/ml mouse recombinant IL-2 (Biolegend, #714604) overnight. These cells were then co-cultured with or without parental tumor irradiated cells (50 Gy) for stimulation in 5:1 ratio of T-cell: tumor-cell in irradiated medium. Protein transport inhibitors, including 1ul/ml Monesin (BD BIOSCIENCES, # 554724) and 1ul/ml Brefeldin A (BD BIOSCIENCES, # 555029) were added into the co-cultures. Cells were incubated for 4 h at 37 °C and transferred to 4 °C overnight. PBMCs isolated from tumor-free C57BL/6 mice were used as a negative control for antigen specificity. The stimulation with αCD3/CD28 beads (Gibco, # 11456D, 1:1 bead-to-cell ratio) was used as a positive control for CD8^+^ Tc cell responses. The levels of IFN-γ, TNF-α, and granzyme B were assessed using a BD LSR Fortessa flow cytometer and analyzed using FlowJo software (Version 10.5.3).

### RNA sequencing

Tumors were collected into RNAlater. RNA was extracted using Qiagen RNAeasy plus mini Kit for three replicates from H_2_0 and Rigosertib treated groups. Samples were submitted to Vanderbilt Technologies for Advanced Genomics (VANTAGE) for RNA QC, library prep and sequencing with a mean of ~ 30 M reads/sample. Samples were sequenced on the Illumina NovaSeq 6000 using 150 bp paired-end reads. Reads were then aligned to the STAR aligner for reads. FASTQ files containing raw reads from the RNA-seq analyses have been deposited with the NCBI GEO under accession GSE149737. Gene set enrichment analyses from the RNA-seq data were performed according to the instructions (https://geneontology.org/).

### Pathology and immunohistochemistry assessment

AST and ALT analysis, complete blood cell count and immunohistochemistry (IHC) analysis on 10% buffered formalin-fixed, paraffin-embedded tissue sections were performed by the Vanderbilt Translational Pathology Shared Resource [51]*.* Slides were placed on the Leica Bond Max IHC stainer. All steps besides dehydration, clearing and coverslipping are performed on the Bond Max. Slides are deparaffinized. Heat induced antigen retrieval was performed on the Bond Max using their Epitope Retrieval 2 solution for 20 min. Slides were incubated with primary antibody (Suppl.Table 3). The Bond Polymer Refine detection system (DS9800, Leica, Buffalo Grove, IL) was used for visualization. Slides were the dehydrated, cleared and coverslipped. IHC slides were scanned at the Vanderbilt Digital Pathology Shared Resource. The automated quantification of the percentages of the target-positive cells was performed by Leica Biosystems’ Digital Image Hub at Vanderbilt, using the software available with the Leica SCN400 Slide Scanner. For multiplex IHC, sequential cycles of IHC staining of antibodies were conducted. The Bond Polymer Refine detection system and the Vector AEC (3-amino-9-ethylcarbazole) HRP Substrate (SK-2405, Vector Lab, Burlingame, CA) was used for visualization. After each cycle of staining, the full slides were immediately scanned and antibodies were stripped from the section, eliminating the risk of antibody cross-reactivity when performing the next round of labelling. Hematoxylin was used in every cycle of staining for cell identification and matching. Computational cell segmentation and images processing were performed by Leica Biosystems’ Digital Image Hub at Vanderbilt. Fiji/ImageJ (version 1.52p) was used for image pseudocoloring and overlay.

### Cytokine array

Tumor lysates, serum, and cell culture supernatant samples were examined in cytokine arrays using Mouse Cytokine Array G3 Kit (62 cytokines) (RayBiotech), per the manufacture’s protocol. For each mouse tissue, samples were pooled from 5 mice per group.

### Cells with stable CD40 knockdown

For specific knockdown of CD40, plasmids (Sigma-Aldrich) of retroviral vectors carrying CD40 shRNA against coding sequence (CDS), untranslated region (UTR) or random sequence as control shRNA were developed to infect HEK293T cells with FugeneHD (Promega). Virus-containing supernatants were used to transduce YUMM3.3 melanoma cells twice, 24 h apart with 6 μg/ml polybrene. Twenty-four hours after the final transduction, stable cells expressing the shRNA were isolated via selection in the presence of 1 μg/ml puromycin (RPI Corp). The expression of CD40 protein in the stable cells was evaluated at mRNA and cell surface protein levels. The cells that showed ≥60% reduction of CD40 were used for experiments.

### Publicly available database analysis

Whole-genome copy number alteration (CNA) analysis, microarray, and RNA sequencing-based gene expression and mutation burden profiling in melanoma (TCGA) were obtained from cBioPortal for Cancer Genomics (www.cbioportal.org). Kaplan–Meier survival curves were used to show the effect of target genes on the outcomes of melanoma patients. For drug sensitivity correlation analysis, human cancer cell line datasets published in Oncomine (www.oncomine.org) was used. For RNA-Seq dataset analysis, two bulk RNA-seq datasets, which studied resistance mechanisms of immune checkpoint blockade [52, 53], were downloaded from the original literature and collected into the Immu-Mela database (https://bioinfo.vanderbilt.edu/database/Immu-Mela/). The association of CD40 expression and immunotherapy response in these two datasets were evaluated and obtained from the Immu-Mela database. The scRNA-seq data and the overall expression of immune resistance score (OE.immune_resistance) were downloaded from Single Cell Portal (https://singlecell.broadinstitute.org/single_cell/study/SCP109/melanoma-immunotherapy-resistance#study-summary) [54]. As described in the original paper [54], gene expression was quantified as log_2_(TPM/10 + 1), where transcripts per million (TPM) was divided by 10 and log2-transformed after the addition of 1. Overall expression of immune resistance (OE.immune_resistance) score was computed for the variation in the signal-to-noise ratio across hallmark genes of immune evasion, suppression, and exclusion in malignant cells [54]. Spearman correlation was used to calculate the association between CD40 expression and the OE.immune_resistance score.

### Statistical analysis

Treatment effects in standard two-group experiments were compared using a two-sample t-test between independent samples or a paired t-test between correlated samples. A one-way analysis of variance (ANOVA) with post hoc Tukey's HSD test was used for more than two-group experiments to compare treatments’ differences. For in vivo experiments, the progression of tumor volume (mm^3^) over time among groups of mice receiving different therapies were compared with a linear mixed-effects regression model to account for within mouse correlation. A square root or a natural log transformation was implemented to better meet the normality assumptions. The likelihood ratio test was performed to identify statistically significant time by treatment effects. A statistically significant interaction implies that the magnitude of treatment differences depends on the actual day of measurement. Using model-based (least-square) means, average tumor growth between treatment groups was estimated and compared with the Wald test. For the synergy test, a mixed-effect model with individual drug effects and the interactive effect over time was fitted to evaluate the interaction between two drugs in tumor growth rate where tumor volume was analyzed on a natural log scale. The adjusted average tumor growth rates were estimated for individual drugs and the combination drug based on the model. The likelihood ratio test was performed to determine if drug interactions were statistically significant (*p* < 0.05). A statistically significant interaction implies the effect of drug combinations is supra-additive (synergistic) when the interaction plot (Fig. 4 e) showed lines are in the same direction, but one is steeper than the other. The Bonferroni correction was used to control the experiment-wise type I error rate at 5%. Residual analysis was performed to evaluate model assumptions. Survival curves are estimated using the Kaplan-Meier method and compared statistically using the log-rank test. For RNA-Seq experiments, pre-filtering of the dataset was conducted by only including genes with a median count of 5 or more in at least one group to increase the speed of the transformation and testing functions within DESeq2 [72]. Raw count data were used in DESeq2, implementing the negative binomial generalized linear model to test for differential gene expression. The Benjamini-Hochberg (BH) approach was used to adjust the *p*-value [73]. Spearman’s rank correlation was used to measure the strength and direction of a monotonic association between two ranked variables. All tests of statistical significance were two-sided. GraphPad’s Prism 8.0.1 software and R language (version 3.3.0.) were used for the statistical analysis. Data are presented with mean with SE. *p ≤ 0.05; **p ≤ 0.01; ***p ≤ 0.001; ****p ≤ 0.0001.

## Supplementary Information

**Additional file 1: Supplementary Figure 1**. Rigosertib promotes cell death, ROS production and inhibits AKT activation as well as mitosis of melanoma cells in vitro.

**Additional file 2: Supplementary Figure. 2**. Rigosertib demonstrates a general anti-tumor effect in mice.

**Additional file 3: Supplementary Figure. 3**. RGS promotes plasminogen activating cascade and synaptic vesicle trafficking in the TME.

**Additional file 4: Supplementary Figure 4**. Rigosertib promotes conventional dendritic cells and T cells in the tumor-draining lymph nodes.

**Additional file 5: Supplementary Figure. 5**. Rigosertib-induced caspase-dependent cell death is important for the anti-tumor responses in vivo.

**Additional file 6: Supplementary Figure. 6.** Rigosertib demonstrates a safe profile in healthy and tumor-bearing mice.

**Additional file 7: Supplementary Figure. 7**. Rigosertib plus ICB treatment induces NK and T cell responses in the tumor draining lymph nodes (TDLN).

**Additional file 8: Supplementary Figure. 8**. No Toxicity of Rigosertib + ICB combinational treatment in vivo.

**Additional file 9: Supplementary Figure. 9**. Cytokine production and antigen (Ag) specificity of tumor-infiltrating CD8^+^ cytotoxic T cells (Tc).

**Additional file 10: Supplementary Figure. 10**. Rigosertib + ICB combinatorial treatment reduces PD-L1^+^ Bregs.

**Additional file 11: Supplementary Figure. 11**. Characterization of CD40 knockdown clones.

**Additional file 12: Supplementary Figure. 12**. CD40 ligand (CD40L) expression in tumor-infiltrating T cells.

**Additional file 13: Supplementary Figure. 13**. Intact adaptive immune system is required for rigosertib to inhibit melanoma tumor growth in vivo.

**Additional file 14: Supplementary Figure. 14**. Copy number of CD40 prognoses sensitivity of human melanoma cells to the RAF inhibitors.

**Additional file 15: Supplementary Figure. 15**. CD80 and ICOSL do not prognose sensitivity of human melanoma cells to the RAF inhibitors.

**Additional file 16: Supplementary Figure. 16**. Bulk tumor RNA-Seq analysis of two datasets with both transcriptomics and immunotherapy response in melanoma

**Additional file 17: Supplementary Table 1**. Genetic background of melanoma models used in this study

**Additional file 18: Supplementary Table 2**. Patient characteristics and response to targeted therapy

**Additional file 19: Supplementary Table 3**. List of antibodies used in western blotting and IHC staining

**Additional file 20: Supplementary Table 4**. List of antibodies used in flow cytometry

## Data Availability

All data generated during this study are included in this published article and its supplementary files. FASTQ files containing raw reads from the RNA-seq analyses have been deposited with the NCBI GEO under accession GSE149737.

## Abbreviations

Ag: Antigen
AiP: Apoptosis-induced proliferation
ALT: Alanine aminotransferase
APCs: Antigen-presenting cells
AST: Aspartate aminotransferase
BRAF: V-raf murine sarcoma viral oncogene homolog B1
Bregs: Regulatory B cells
CD: Cluster of differentiation
CD40L: CD40 ligand
cDC: Conventional dendritic cells
CDS: Coding sequence
CTLA-4: Cytotoxic T-lymphocyte-associated protein 4
DC: Dendritic cells
ER: Endoplasmic reticulum
FOB: Follicular B cell
GzmB: Granzyme B
HDAC: Histone deacetylase
ICB: Immune checkpoint blockade
ICD: Immunogenic cell death
ICOS: Inducible T-cell costimulator
IFN: Interferon
IL: Interleukin
MDS: Myelodysplastic syndrome
MHC: Major histocompatibility complex
NRAS: Neuroblastoma RAS viral oncogene homolog
OS: Overall survival
PD-1: Programmed cell death protein 1
pDC: Plasmacytoid dendritic cells
PD-L1: Programmed cell death protein 1 ligand 1
PDX: Patient-derived xenografts
PFS: Progression free survival
PI3K: Phosphoinositide 3-kinase
PLK1: Polo-like kinase 1
RBD: RAS-binding domain
RGS: Rigosertib
ROS: Reactive oxygen species
sc: Single cell
Tc: Cytotoxic T
TCGA: The cancer genome atlas
TDLN: Tumor-draining lymph node
Th: T helper
TME: Tumor microenvironment
TNF: Tumor necrosis factor
Tregs: Regulatory T cells
UTR: 3′ untranslated region
