# Supplementary material for: Novel induction of CD40 expression by tumor cells with RAS/RAF/PI3K pathway inhibition augments response to checkpoint blockade
Source: Mol Cancer. 2021 Jun 6;20:85. doi: 10.1186/s12943-021-01366-y (PMC8182921; doi:10.1186/s12943-021-01366-y)
Supplement: Supplementary file 1 [file 12943_2021_1366_MOESM10_ESM.docx]

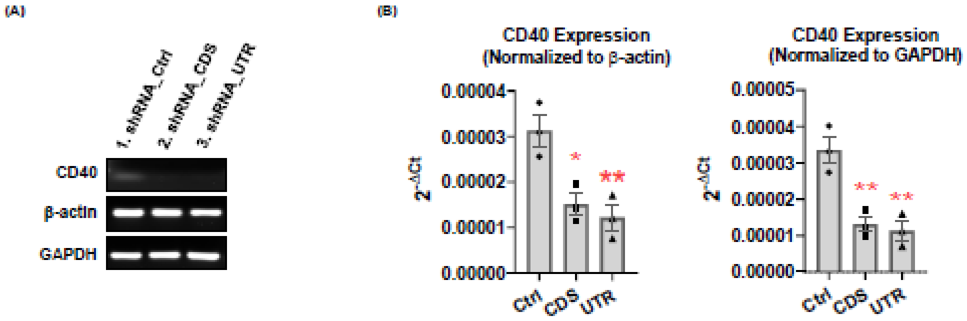

Supplementary Figure 10. Characterization of CD40 knockdown clones. YUMM3.3 cells were transduced with retroviral vectors containing shRNAs against coding sequence (CDS), untranslated region (UTR) or random sequences (Ctrl). CD40 expression by different knockdowns was determined by (A) RT-PCR and (B) qRT-PCR.
