# Supplementary material for: Novel induction of CD40 expression by tumor cells with RAS/RAF/PI3K pathway inhibition augments response to checkpoint blockade
Source: Mol Cancer. 2021 Jun 6;20:85. doi: 10.1186/s12943-021-01366-y (PMC8182921; doi:10.1186/s12943-021-01366-y)
Supplement: Supplementary file 2 [file 12943_2021_1366_MOESM11_ESM.docx]

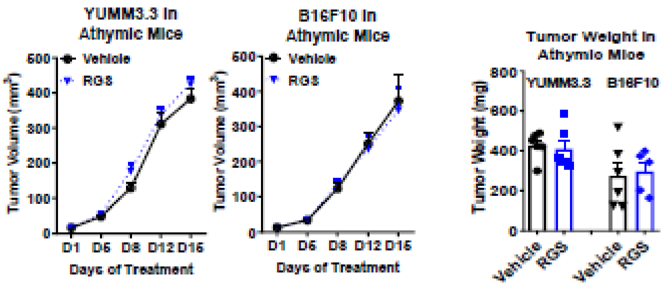

Supplementary Figure 11. Intact adaptive immune system is required for rigosertib to inhibit melanoma tumor growth *in vivo*. Tumor volume and weight of YUMM3.3 cells and B16-F10 cells in athymic female mice. Daily RGS treatment starts at Day 10 post tumor cell inoculation.
