# Supplementary material for: Novel induction of CD40 expression by tumor cells with RAS/RAF/PI3K pathway inhibition augments response to checkpoint blockade
Source: Mol Cancer. 2021 Jun 6;20:85. doi: 10.1186/s12943-021-01366-y (PMC8182921; doi:10.1186/s12943-021-01366-y)
Supplement: Supplementary file 3 [file 12943_2021_1366_MOESM12_ESM.docx]

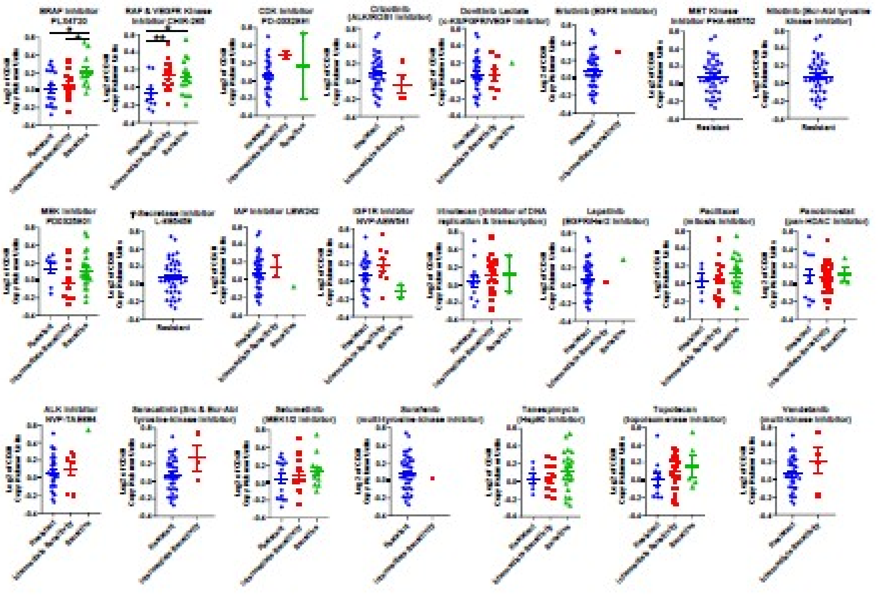

Supplementary Figure 12. Copy number of CD40 prognoses sensitivity of human melanoma cells to the RAF inhibitors. Drug sensitivity analysis of Barretina CellLine 2 (DNA) dataset in Oncomine database.
