# Supplementary material for: Novel induction of CD40 expression by tumor cells with RAS/RAF/PI3K pathway inhibition augments response to checkpoint blockade
Source: Mol Cancer. 2021 Jun 6;20:85. doi: 10.1186/s12943-021-01366-y (PMC8182921; doi:10.1186/s12943-021-01366-y)
Supplement: Supplementary file 4 [file 12943_2021_1366_MOESM13_ESM.docx]

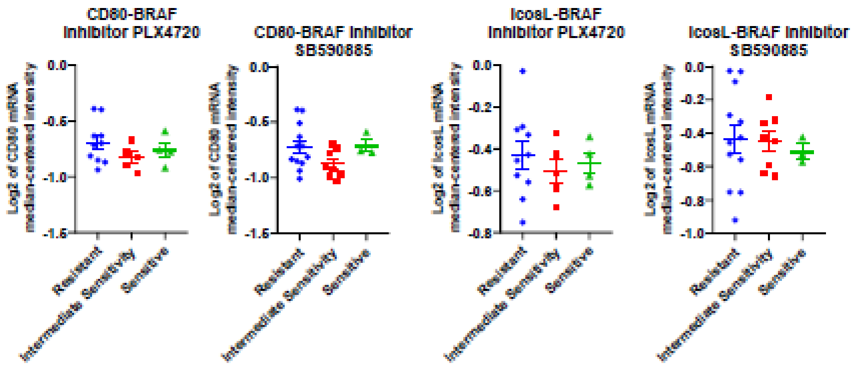

Supplementary Figure 13. CD80 and ICOSL do not prognose sensitivity of human melanoma cells to the RAF inhibitors.
Drug sensitivity analysis Garnett CellLine (mRNA) datasets in Oncomine database.
