# Supplementary material for: Novel induction of CD40 expression by tumor cells with RAS/RAF/PI3K pathway inhibition augments response to checkpoint blockade
Source: Mol Cancer. 2021 Jun 6;20:85. doi: 10.1186/s12943-021-01366-y (PMC8182921; doi:10.1186/s12943-021-01366-y)
Supplement: Supplementary file 5 [file 12943_2021_1366_MOESM14_ESM.docx]

Supplementary Table 1. Genetic background of melanoma models used in this study.

Name	Species	NRAS	BRAF	PTEN	TP53	CDKN2A
HS294T	Human	WT	V599E	-	++ WT	-
SKMel5	Human	WT	V599E	+ WT	++ WT	-
SKMel2	Human	Q61R	WT	+ WT	+++ G245S	+
A375	Human	WT	V600E	+ WT	+ WT	+
B16 F10	Mouse	WT	WT	WT	WT	-
YUMM 4.1	Mouse	WT	WT	-	WT	-
YUMM 5.2	Mouse	WT	V600E	WT	-	WT
YUMM 2.1	Mouse	WT	V600E	-	WT	+/-
YUMM 3.3	Mouse	WT	V600E	WT	WT	-
PDX1179	Human	Q61H	WT	WT	WT	WT
PDX1214	Human	Q61L	WT	WT	Mutant	N/A
PDX2132	Human	WT	V600E	Mutant	Mutant	N/A
PDX1839	Human	WT	V600E	Stop Lost	WT	WT
PDX2195	Human	WT	V600E	WT	WT	WT
PDX2252	Human	WT	V600M	Stop Lost	Stop Lost	Stop Lost
PDX2552	Human	WT	V600E	T398S	WT	Stop Lost
PDX1577	Human	WT	V600E	T398S	WT	WT

Note: WT, wildtype. PDX, patient derived xenografts. N/A, not applicable.
