# Supplementary material for: Novel induction of CD40 expression by tumor cells with RAS/RAF/PI3K pathway inhibition augments response to checkpoint blockade
Source: Mol Cancer. 2021 Jun 6;20:85. doi: 10.1186/s12943-021-01366-y (PMC8182921; doi:10.1186/s12943-021-01366-y)
Supplement: Supplementary file 7 [file 12943_2021_1366_MOESM16_ESM.docx]

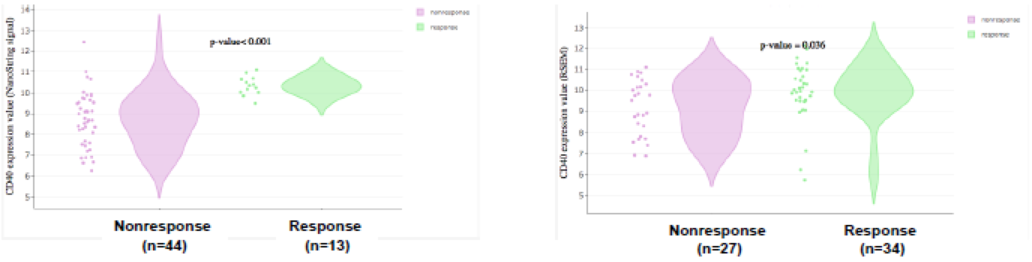

Initially αCTLA4 followed by αPD-1 at progression

αCTLA4 (ipilimumab) or αPD-1 (nivolumab)

Supplementary Figure 16. Bulk tumor RNA-Seq analysis of two datasets with both transcriptomics and immunotherapy response in melanoma.
References: i) Cancer Discov. 2016 Aug;6(8):827-37. doi: 10.1158/2159-8290.CD-15-1545. ii) Cell. 2017 Nov 2;171(4):934-949.e16. doi: 10.1016/j.cell.2017.09.028.
