# Supplementary material for: Novel induction of CD40 expression by tumor cells with RAS/RAF/PI3K pathway inhibition augments response to checkpoint blockade
Source: Mol Cancer. 2021 Jun 6;20:85. doi: 10.1186/s12943-021-01366-y (PMC8182921; doi:10.1186/s12943-021-01366-y)
Supplement: Supplementary file 8 [file 12943_2021_1366_MOESM17_ESM.docx]

Supplementary Table 2. Patient characteristics and response to targeted therapy.

ID	Age	Sex	Mutation	Prior Systemic Therapy	Targeted Therapy	Reason for Discontinuation	Time on Therapy (months)	PFS on BRAF pathway inhibition (months)
0848	48	M	V600E	interferon	D+T	cardiotoxicity	8.5	38.8
					V	skin toxicity	7.1	
					D	PD	38.8	
0880	47	M	V600E	none	V	PD	4.2	4.2
1358	41	F	V600E	ipi/nivo	D+T	PD	8.5	8.5
0923	41	M	V600E	none	D+T	PD	13.3	13.3
1218	60	F	V600E	none	D	MR	3.7	3.7
1262	21	F	V600E	ipi	V	PD	3.5	3.5
1351	62	F	V600E	nivolumab temozolomide	T	PD	1.9	9.8
					D	MR	2.8	
					D+T	PD	9.8	
1441	47	M	V600E	none	V + PX866 (PI3Ki)	PD	15.4	45.1
					D	PD	45.1	
1446	77	F	V600K	none	V	PD	2.3	2.3
1577	44	F	V600E	none	D+T	PD	20.5	20.5
2132	67	M	V600E	ipi/nivo	D+T	PD	14.1	14.1

All metastatic melanoma patients treated at Vanderbilt University Medical Center, 2010-2018. Age, years. Sex, M (male), F (female). Mutation refers to BRAF status as determined by CLIA-approved genetic testing. Prior systemic therapy, ipi (ipilimumab), nivo (nivolumab). Targeted therapy, D (dabrafenib), T (trametinib), V (vemurafenib). Multiple lines of targeted therapy in an individual patient listed on separate lines. Reason for discontinuation, PD (progressive disease), MR (mixed response). PFS (progression free survival), months. Calculated for each line of treatment with targeted therapy based on RECIST criteria and clinician review.
