# Supplementary material for: Novel induction of CD40 expression by tumor cells with RAS/RAF/PI3K pathway inhibition augments response to checkpoint blockade
Source: Mol Cancer. 2021 Jun 6;20:85. doi: 10.1186/s12943-021-01366-y (PMC8182921; doi:10.1186/s12943-021-01366-y)
Supplement: Supplementary file 10 [file 12943_2021_1366_MOESM19_ESM.docx]

Supplementary Table 3. List of antibodies used in western blotting and IHC staining.

Target-Format	Clone	Vendor (CAT#)	Reactivity	Application
Raptor	24C12	Cell Signaling (2280)	H, M	WB
Rictor	53A2	Cell Signaling (2114)	H, M	WB
P-AKT(Ser473)	Polyclonal	Cell Signaling (9271)	H, M	WB
P-AKT(Thr308)	Polyclonal	Cell Signaling (9275)	H, M	WB
AKT	C67E7	Cell Signaling (4691)	H, M	WB
P-JNK	G9	Cell Signaling (9255)	H, M	WB
JNK	Polyclonal	Cell Signaling (9252)	H, M	WB
P-ERK	D13.14.4E	Cell Signaling (4370)	H, M	WB
ERK	137F5	Cell Signaling (4695)	H, M	WB
HSP90	C45G5	Cell Signaling (4877)	H, M	WB
P-PLK1	Polyclonal	Cell Signaling (5472)	H	WB
PLK1	208G4	Cell Signaling (4513)	H	WB
FOX M1	D12D5	Cell Signaling (5436)	H	WB
Cleaved-Caspase3	5A1E	Cell Signaling (9664)	H, M	WB
Caspase-3	Polyclonal	Cell Signaling (9662)	H, M	WB
CD40	Polyclonal	Abcam (ab13545)	H, M	IHC
CD80	EPR1157(2)	Abcam (ab134120)	H	IHC
CD11c	5D11	Leica (PA0554)	H	IHC
CD8	C8/144B	Thermo (MS-457-R7)	H	IHC
SOX10	Polyclonal	Cell Marque (PA0813)	H	IHC
CD3	SP7	Abcam (ab16669)	H, M	IHC
CD8	4SM-15	Ebiosciences (14-0808-80)	M	IHC
B220 (CD45R)	RA3-6B2	BD Pharmigen (553086)	H, M	IHC
CD31	S231	Dianova (DIA310)	M	IHC
Ki67	D3B5	Cell Signaling (122025)	H, M	IHC
FoxP3	FJK-16S	Ebioscience (13-5773-82)	H, M	IHC
Cleaved-Caspase3	5AIE	Cell Signaling (9664S)	H, M	IHC

H, human. M, mouse. WB, western blotting. IHC, immunohistochemistry.
