# Supplementary material for: Novel induction of CD40 expression by tumor cells with RAS/RAF/PI3K pathway inhibition augments response to checkpoint blockade
Source: Mol Cancer. 2021 Jun 6;20:85. doi: 10.1186/s12943-021-01366-y (PMC8182921; doi:10.1186/s12943-021-01366-y)
Supplement: Supplementary file 11 [file 12943_2021_1366_MOESM1_ESM.docx]

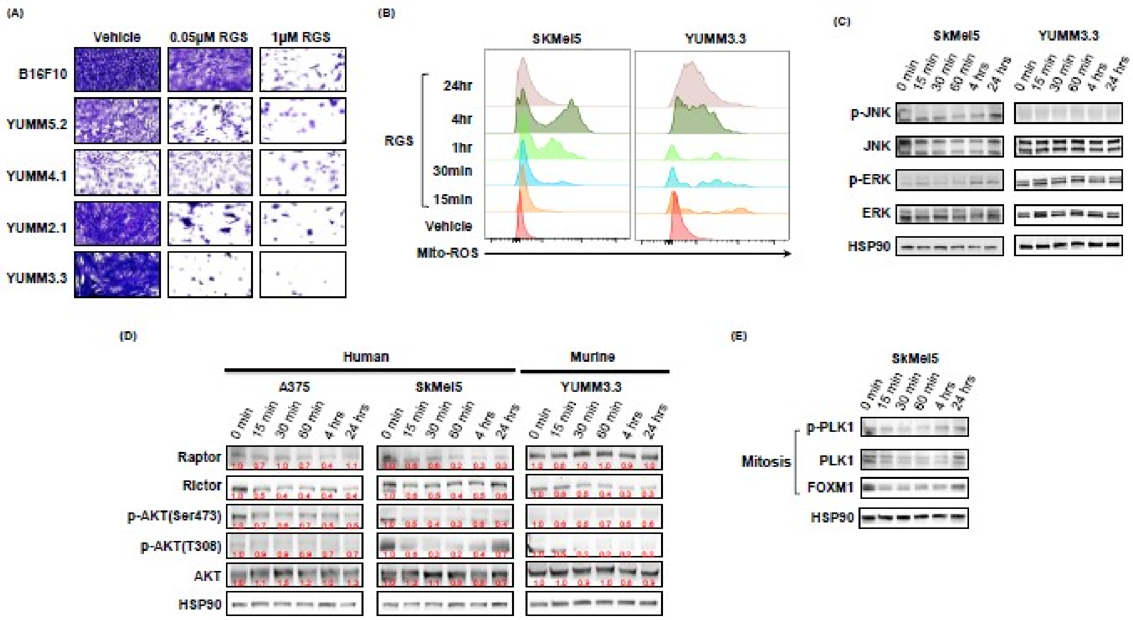

Supplementary Figure 1. Rigosertib promotes cell death, ROS production and inhibits AKT activation as well as mitosis of melanoma cells *in vitro*. (A) Melanoma cell viability detected by Crystal Violet assay at 72hrs post RGS treatment. Cells were seeded in 6-well plate with 100,000 cells per well. (B) Mitochondria ROS production measured by 1uM MitoSOX stain using flow cytometry in response to 100uM RGS. (C-E) Whole-cell extracts were harvested and immunoblotted to detect phosphorylated and total proteins. HSP90 was used as a loading control for densitometry quantification (red numbers). Data are representative of at least two independent experiments.
