# Supplementary material for: Novel induction of CD40 expression by tumor cells with RAS/RAF/PI3K pathway inhibition augments response to checkpoint blockade
Source: Mol Cancer. 2021 Jun 6;20:85. doi: 10.1186/s12943-021-01366-y (PMC8182921; doi:10.1186/s12943-021-01366-y)
Supplement: Supplementary file 12 [file 12943_2021_1366_MOESM20_ESM.docx]

Supplementary Table 4. List of antibodies used in flow cytometry

Target	Conjugate	Clone	Vendor (CAT#)
CD45	APC/Cy7	30-F11	BioLegend (103116)
Ly6G	PE/Cy7	1A8	BioLegend (127618)
CD11c	BV421	N418	BioLegend (117330)
F4/80	APC	BM8	BioLegend (123115)
CD11b	Alexa488	M1/70	BioLegend (101217)
Ly6C	BV605	HK1.4	BioLegend (128035)
MHCII	AlexaFlour700	M5/114.15.2	BioLegend (107621)
CD206	PE	C068C2	BioLegend (141705)
CD8	PerCP-Cy5.5	53-6.7	BioLegend (100734)
CD103	BV785	M290	BD Horizon (564322)
CD3	PerCP/eFluor 710	17A2	eBioscience (460032-80)
CD4	BV421	GK1.5	BioLegend (100438)
CD8	AlexaFlour700	53-6.7	BioLegend (100729)
NK1.1	PE	PK136	BioLegend (108708)
CD69	APC	H1.2F3	BioLegend (104513)
CD25	PE/Cy7	PC61	BioLegend (102016)
TIM3	BV605	RMT3-23	BioLegend (119721)
LAG3	BV785	C9B7W	BioLegend (125219)
CD5	BV421	53-7.3	BioLegend (100629)
CD19	VioBright FITC	6D5	Miltenyi Biotec (130-105-171)
CD21	PerCP-Cy5.5	7E9	BioLegend (123416)
CD23	APC	B3B4	BioLegend (101619)
B220	AlexaFlour700	RA3-6B2	BD Pharmingen (557957)
CD43	PE	1B11	BioLegend (121208)
PD-L2	PE/Cy7	TY25	BioLegend (107214)
PD-L1	BV785	10F.9G2	BioLegend (124331)
CD80	BV421	16-10A1	BioLegend (104725)
CD86	BV650	GL-1	BioLegend (105036)
CD40	FITC	HM40-3	BioLegend (102905)
ICOSL	PE	HK5.3	BioLegend (107405)
MHCI	PerCP/eFluor 710	AF6-88.5.5.3	eBioscience (46-5958-80)
Ki67	FITC	7B11	Invitrogen (334711)
Foxp3	PerCP-Cy5.5	FJK-16s	Invitrogen (45-5773-82)
CD44	BV650	IM7	BioLegend (103049)
CD62L	BV570	MEL-14	BioLegend (104433)
