# Supplementary material for: Novel induction of CD40 expression by tumor cells with RAS/RAF/PI3K pathway inhibition augments response to checkpoint blockade
Source: Mol Cancer. 2021 Jun 6;20:85. doi: 10.1186/s12943-021-01366-y (PMC8182921; doi:10.1186/s12943-021-01366-y)
Supplement: Supplementary file 13 [file 12943_2021_1366_MOESM2_ESM.docx]

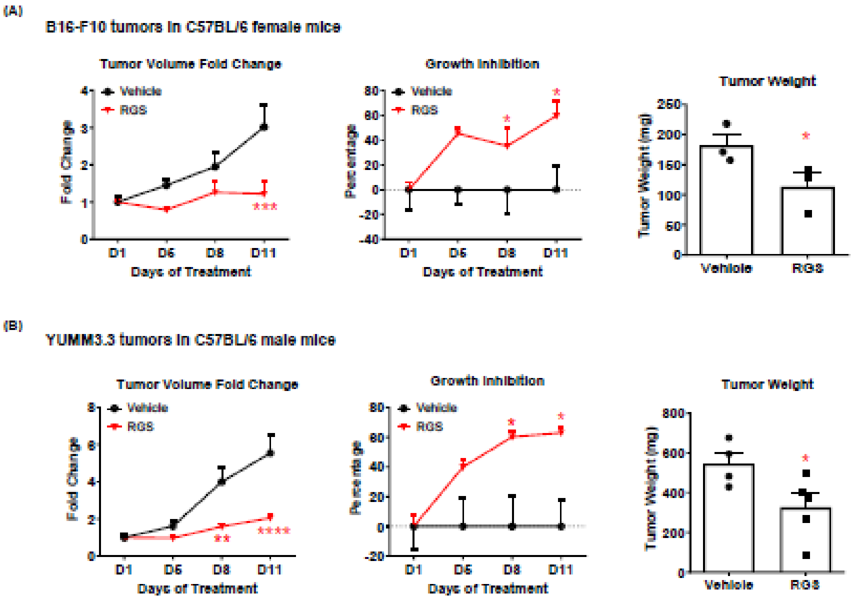

Supplementary Figure 2. Rigosertib demonstrates a general anti-tumor effect in mice. Tumor volume fold change, weight and tumor growth inhibition percentage of (A) B16-F10 tumors in C57BL/6 female mice and (B) YUMM3.3 cells in C57BL/6 male mice. Daily RGS treatment starts at Day 10 post tumor cell inoculation. Tumor volume fold change was normalized to the volume before RGS treatment at day 10 post inoculation. $^{*}p \le 0.05$; $^{**}p \le 0.01$; $^{****}p \le 0.0001$; statistical analysis was compared with the vehicle control.
