# Supplementary material for: Novel induction of CD40 expression by tumor cells with RAS/RAF/PI3K pathway inhibition augments response to checkpoint blockade
Source: Mol Cancer. 2021 Jun 6;20:85. doi: 10.1186/s12943-021-01366-y (PMC8182921; doi:10.1186/s12943-021-01366-y)
Supplement: Supplementary file 14 [file 12943_2021_1366_MOESM3_ESM.docx]

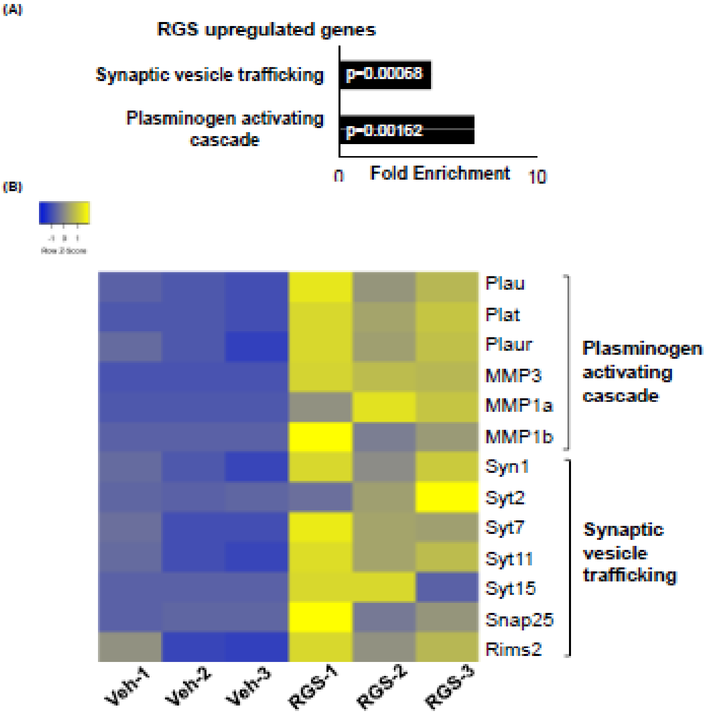

Supplementary Figure 3. RGS promotes plasminogen activating cascade and synaptic vesicle trafficking in the TME. (A) Gene ontology enrichment analysis of gene targets upregulated in YUMM3.3 tumors post 17 days of RGS treatment. Displaying only results for Fold Enrichment >3 and FDR $p < 0.05$. (B) Heatmap summarizes 13 pathway-associated targets from a total of 1311 genes screened that were significantly upregulated due to RGS treatment. Statistical analysis was compared with the vehicle (H_2O) control.
