# Supplementary material for: Novel induction of CD40 expression by tumor cells with RAS/RAF/PI3K pathway inhibition augments response to checkpoint blockade
Source: Mol Cancer. 2021 Jun 6;20:85. doi: 10.1186/s12943-021-01366-y (PMC8182921; doi:10.1186/s12943-021-01366-y)
Supplement: Supplementary file 15 [file 12943_2021_1366_MOESM4_ESM.docx]

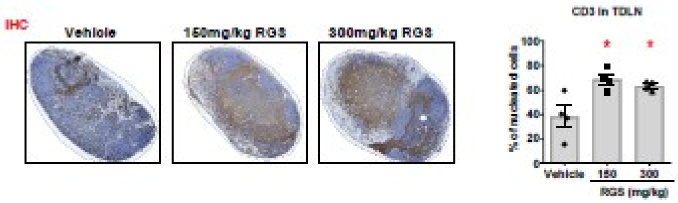

Supplementary Figure 4. Rigosertib promotes conventional dendritic cells and T cells in the tumor-draining lymph nodes. 0.3 million YUMM3.3 cells per flank were inoculated in C57BL/6 female mice. Daily RGS treatment starts at Day 10 post tumor cell inoculation. IHC analysis of immune profiles in the tumor-draining lymph nodes. *$p \leq 0.05$; statistical analysis was compared with the vehicle control.
