# Supplementary material for: Novel induction of CD40 expression by tumor cells with RAS/RAF/PI3K pathway inhibition augments response to checkpoint blockade
Source: Mol Cancer. 2021 Jun 6;20:85. doi: 10.1186/s12943-021-01366-y (PMC8182921; doi:10.1186/s12943-021-01366-y)
Supplement: Supplementary file 16 [file 12943_2021_1366_MOESM5_ESM.docx]

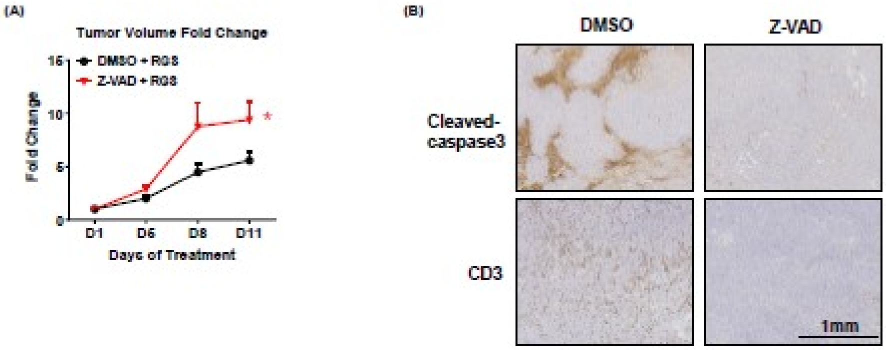

Supplementary Figure 5. Rigosertib-induced caspase-dependent cell death is important for the anti-tumor responses *in vivo*. (A) Tumor volume fold change of YUMM3.3 cells in female C57BL/6 mice. RGS (300 mg/kg, daily, oral), Z-VAD (2 mg/kg, daily, IP) and DMSO vehicle treatments start at Day 10 post tumor cell inoculation. (B) Representative images of cleaved-caspase-3 and CD3 protein levels observed from day 11 post RGS+/-Z-VAD treatment of YUMM3.3 tumors by immunohistochemistry. *$p \leq 0.05$; statistical analysis was compared with the vehicle DMSO control + RGS.
