# Supplementary material for: Novel induction of CD40 expression by tumor cells with RAS/RAF/PI3K pathway inhibition augments response to checkpoint blockade
Source: Mol Cancer. 2021 Jun 6;20:85. doi: 10.1186/s12943-021-01366-y (PMC8182921; doi:10.1186/s12943-021-01366-y)
Supplement: Supplementary file 17 [file 12943_2021_1366_MOESM6_ESM.docx]

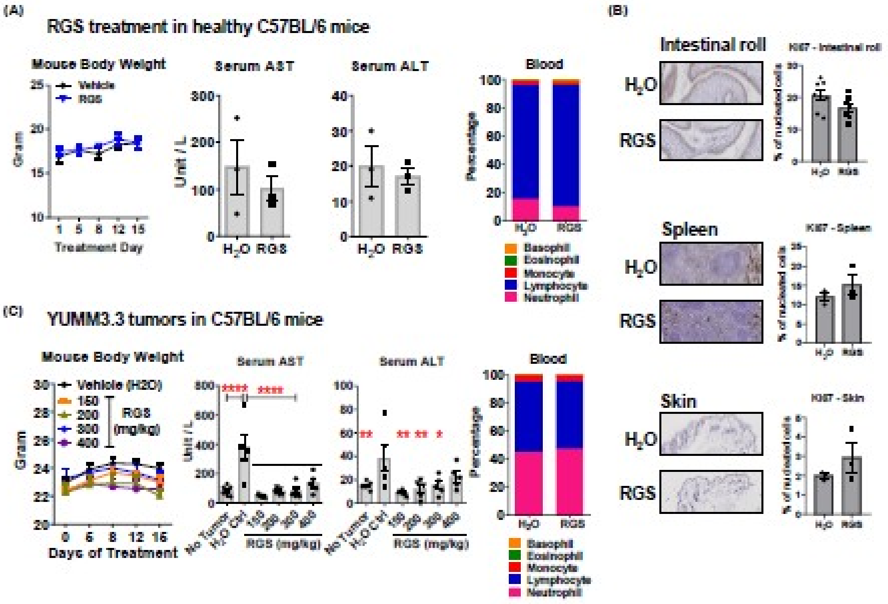

Supplementary Figure 6. Rigosertib demonstrates a safe profile in healthy and tumor-bearing mice. Mouse body weight, serum AST/ALT level and complete blood cell count of (A) tumor-free and (C) YUMM3.3 tumor-bearing C57BL/6 mice under 15 days of rigosertib treatment. (B) Ki67 IHC stain of tissues from tumor-free C57BL/6 mice under 15 days of rigosertib treatment. *$p \le 0.05$; **$p \le 0.01$; ****$p \le 0.0001$; statistical analysis was compared with the vehicle control.
