# Supplementary material for: Novel induction of CD40 expression by tumor cells with RAS/RAF/PI3K pathway inhibition augments response to checkpoint blockade
Source: Mol Cancer. 2021 Jun 6;20:85. doi: 10.1186/s12943-021-01366-y (PMC8182921; doi:10.1186/s12943-021-01366-y)
Supplement: Supplementary file 18 [file 12943_2021_1366_MOESM7_ESM.docx]

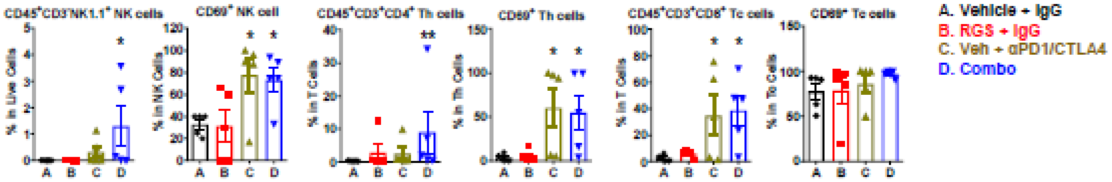

Supplementary Figure 7. Rigosertib plus ICB treatment induces NK and T cell responses in the tumor draining lymph nodes (TDLN). YUMM3.3 cells were injected in C57BL/6 female mice. Treatment starts at Day 10 post tumor cell inoculation. Analysis of indicated immune markers by flow cytometry of single cell suspensions prepared from TDLNs at day 17 post-treatment.
