# Supplementary material for: Novel induction of CD40 expression by tumor cells with RAS/RAF/PI3K pathway inhibition augments response to checkpoint blockade
Source: Mol Cancer. 2021 Jun 6;20:85. doi: 10.1186/s12943-021-01366-y (PMC8182921; doi:10.1186/s12943-021-01366-y)
Supplement: Supplementary file 19 [file 12943_2021_1366_MOESM8_ESM.docx]

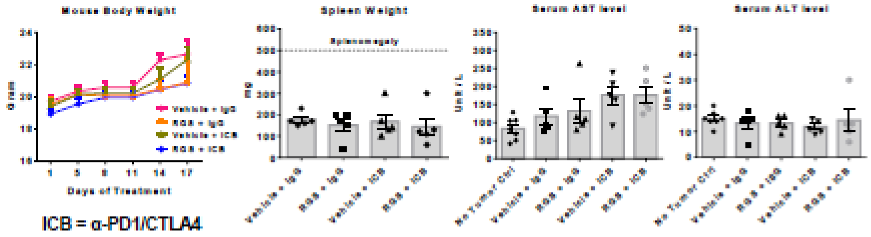

Supplementary Figure 8. No Toxicity of Rigosertib + ICB combinational treatment *in vivo*. Mouse body weight, spleen weight as well as serum alanine aminotransferase (ALT) and aspartate aminotransferase (AST) of YUMM3.3 tumor-bearing C57BL/6 mice under 17 days of treatment. ICB, immune checkpoint blockade (αPD1 and αCTLA4).
