# Supplementary material for: Novel induction of CD40 expression by tumor cells with RAS/RAF/PI3K pathway inhibition augments response to checkpoint blockade
Source: Mol Cancer. 2021 Jun 6;20:85. doi: 10.1186/s12943-021-01366-y (PMC8182921; doi:10.1186/s12943-021-01366-y)
Supplement: Supplementary file 20 [file 12943_2021_1366_MOESM9_ESM.docx]

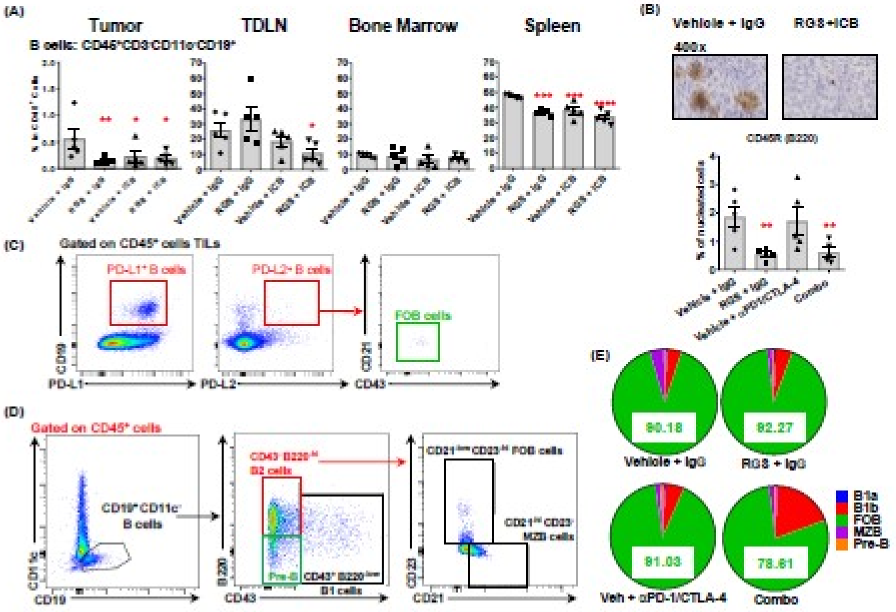

Supplementary Figure 9. Rigosertib + ICB combinatorial treatment reduces PD-L1+ Bregs. 0.3 million YUMM3.3 cells per flank were inoculated in C57BL/6 female mice. Treatment starts at Day 10 post tumor cell inoculation. (A,C-E) Flow cytometric and (B) IHC analysis of immune profiles. TDLN, tumor-draining lymph nodes. ICB, immune checkpoint blockade (αPD1 and αCTLA4).
